# Dietary Recommendations for Body Mass and Composition Manipulation in Male and Female Athletes: a Scoping Review of Consensus Statements, Position Stands and Practice Guidelines from International Expert Groups

**DOI:** 10.1007/s40279-025-02285-4

**Published:** 2025-08-21

**Authors:** Lauren V. Delany, Nessan Costello, Ben Jones, Susan H. Backhouse

**Affiliations:** 1 Carnegie School of Sport, Leeds Beckett University, Headingley Campus, Leeds, LS6 3QU UK; 2 Sale Sharks Rugby Club, Manchester, UK; 3 England Performance Unit, Rugby Football League, Manchester, UK; 4 Division of Physiological Sciences, Department of Human Biology, Faculty of Health Sciences, The University of Cape Town and the Sports Science Institute of South Africa, Cape Town, South Africa; 5 Premiership Rugby Limited, London, UK

## Abstract

**Background:**

Body mass and composition (fat and fat-free mass) manipulation is a common practice in sport, yet it can pose significant risks to athlete health and wellbeing. Practitioners must continually adapt to the growing body of evidence to implement safe, effective and context-specific practice.

**Objective:**

This scoping review aimed to summarise dietary recommendations for altering body mass or composition in male and female, adult non-disabled athletes and appraise how these expert-group led recommendations have evolved over time.

**Methods:**

Electronic databases, including SCOPUS, PubMed, SPORTDiscus, CINAHL Complete and APA PsycINFO were searched (last search 2 August 2024) without date restrictions. Papers were included if they provided dietary recommendations for altering body mass or composition in adult non-disabled athlete populations and were published by an expert organisation.

**Results:**

From 6068 records screened, 73 documents were included, comprising 45 consensus statements, 27 position stands and 1 practice guideline, endorsed by 14 organisations and developed by 328 experts from 25 countries. Athletics (*n* = 19), aquatics (*n* = 7) and team sports (*n* = 5) were the most represented, leaving many sports underrepresented. A total of 50 documents were standalone rather than part of an updated series. Only 40 papers addressed specific targets, rates or timing of outcome changes. Individualised, realistic and health-focussed targets were recommended, aligned with the athlete’s sport, position, sex, age and competition phase, with gradual changes (e.g. 0.5–1.0 kg/week fat loss) to enhance performance. Common strategies for altering body mass and composition included creating an energy surplus (500–1000 kcal/day) or deficit (250–1000 kcal/day), maintaining energy availability above 30 kcal/kg fat-free mass/day, and periodising carbohydrate intake (3–12 g/kg/day) on the basis of training demands. Protein intake (1.6–2.4 g/kg/day) was recommended across 4–6 feeds from high-quality sources, alongside targeted supplements such as creatine, whey protein and a multi-vitamin and mineral. Recommendations focussed minimal attention on nutrients such as fats, fibre or micronutrients, and the language used was often vague, leaving significant room for interpretation.

**Conclusions:**

Developing sport-specific, behaviourally anchored and regularly updated dietary recommendations, informed by athlete and multidisciplinary team input, is recommended. This approach would provide actionable, athlete-centred strategies that effectively support body composition goals whilst prioritising health, wellbeing and performance. OSF Registration https://doi.org/10.17605/OSF.IO/B4YJT

**Supplementary Information:**

The online version contains supplementary material available at 10.1007/s40279-025-02285-4.

## Key Points

**Table:** 

1. Most recommendations emphasised the importance of safety in practice: engage a qualified sport dietitian or nutritionist; individualise outcome and dietary targets; adhere to basic dietary principles, such as adjusting calorie and protein intakes, with most supplements deemed unnecessary; and ensure a minimum energy availability target of 30 kcal/kg fat-free mass/day to support athlete health.
2. This review highlighted that body composition recommendations are rarely specified in precise behavioural terms, such as specifying what, who, when, where and how (using active verbs). An opportunity to embed a more behavioural approach was identified, making recommendations more practical and implementable for both athletes and practitioners.
3. Developing recommendations for body mass or composition manipulation is only one step in enabling a healthy environment for athletes to achieve their athletic pursuits. Further research on the extent to which such recommendations are put into practice and what factors influence their implementation would further advance sport and exercise nutrition policy and practice.

## Introduction

Body mass and body composition (fat and fat-free mass) manipulation are widely practiced in sport, but do not always prioritise athlete health and wellbeing. Such practices are used to monitor physical changes [1], assess training interventions [2, 3] and evaluate dietary intake [4]. In some sports, manipulating body composition can influence performance involvement (e.g. making weight in weight-category sports [5, 6]), improve underlying performance indicators (e.g. increasing power:mass ratio to augment running economy [7]) or directly impact performance outcomes (e.g. increasing body mass to support collision dominance in contact sports [8]). However, the current practice of athlete body mass and composition manipulation has raised health and wellbeing concerns [3, 9–11]. For example, athletes have reported inappropriate body monitoring and body shaming, leading to eating disorders [12, 13], whilst others highlight the use of prohibited substances to alter body mass, fat and fat-free mass [14]. Over the past decade, practitioners in elite environments have become increasingly concerned about the emphasis placed on body composition, which may heighten the risk of body dissatisfaction, disordered eating, eating disorders and problematic low energy availability, leading to relative energy deficiency in sport (REDs) [15]. The need to create safer sporting environments that prioritise athlete health, performance and welfare has therefore been called for [3, 13, 16]. Within such environments, the implementation of evidence-based guidelines that give equal weighting to athlete health, wellbeing and performance is required.

The International Olympic Committee have recently published best practice recommendations for the process of body composition assessment and monitoring in sport to reduce health and performance risks [15]. These recommendations provide professional guidelines on the process prior to dietary intervention, including assessment justification, consent, method selection, data capture and interpretation, reporting and appropriate communication and monitoring [15]. It is also advised that the multidisciplinary support team involved in this process should include as a minimum a qualified, experienced sports dietitian/nutritionist, sports physiologist/strength coach, psychologist and sports medicine physician [15]. A key role of the sport nutritionist/dietitian in this context is to advise on dietary interventions to manipulate body mass or composition, applying recommendations from sport or nutrition governing bodies, institutes of sport or nutrition and sport councils [17–19]. As such, guidance documents serve as crucial tools for informed decision-making in the sporting environment and ensuring athlete health and wellbeing.

Given the wide range of sports and the numerous expert organisations developing guidance documents, a comprehensive summary of dietary recommendations to manipulate body mass or composition, applicable to male and female adult athletes, is required. Scoping reviews are an ideal tool to determine the scope and coverage of existing literature, providing insights into the volume, focus and extent of research on a topic [20, 21]. A scoping review can then act as a precursor to a sporting organisation conducting their own systematic review to produce bespoke recommendations for that sport. Consequently, the objective of this scoping review is to identify and collate all dietary recommendations from expert groups for altering body mass or composition in athletes and to examine how recommendations have changed over time. Secondly, this review aims to identify and analyse gaps in the literature to inform future research.

## Methods

The methodology for this scoping review was conducted on the basis of the five stages outlined by Arksey and O’Malley [22], including the updated framework by Levac et al. [23] and methodological guidance from the Joanna Briggs Institute [21, 24]. The Preferred Reporting Items for Systematic Reviews and Meta-analysis extension for Scoping Reviews (PRISMA-ScR) guidelines and checklist were followed for reporting the results [25]. All items of the reporting guidelines were included (see supplementary information 1). A critical appraisal (items 12 and 16) was not conducted, as this is optional in scoping reviews, and our purpose was to provide an overview of the existing evidence regardless of methodological quality or risk of bias [25]. A review protocol was developed prior to commencing this study and registered online on Open Science Framework (10.17605/OSF.IO/B4YJT).

### Stage 1: Identifying the Research Question

The following research question was identified, considering the population, concept and context:

*What are the dietary recommendations from expert groups for altering body mass or composition (including fat mass and fat-free mass) in male and female adult non-disabled athletes across all sports?*

### Stage 2 Identifying Relevant Studies

#### Inclusion Criteria

Inclusion criteria included peer-reviewed papers from any country and published in the English language. Papers were eligible for inclusion if they provided dietary recommendations for altering body mass or composition at any level of athletic populations (tiers 1–5 [26]). Papers with recommendations for both male and female athletes over 18 years of age were included. Body composition was defined as fat mass and fat-free mass following the definitions of Wang et al. [27] and Buckinx et al. [28], as these components are measurable by practitioners and typically implied when targeting change. However, due to the heterogeneity and inconsistency of language used in the literature to describe fat-free mass, all synonyms were included, for example, muscle mass, lean mass or muscle protein. Similarly, all synonyms for the process of increasing (e.g. gain, build, grow) body or fat-free mass, or decreasing (e.g. drop, lose, reduce) body or fat mass were included.

Papers were included if they were endorsed by an established group or organisation that had practitioner or research expertise in nutrition or dietetics, medicine, sport science or sport. Organisations included for example, sport governing bodies, nutrition/dietetic professional bodies, institutes of sport or nutrition, sport councils and Olympic Associations. These organisations were responsible for defining expertise of those involved in recommendation development. Policy documents encompassed practice guidelines, consensus statements (or review papers from consensus conferences) and position stands.

#### Exclusion Criteria

Papers were excluded if they had an incompatible study design, for example, validation or comparison studies, cross-sectional or intervention studies, literature or narrative review, systematic or scoping reviews, meta-analysis, abstracts, book chapters and conference proceeding. Papers were also excluded if they only included recommendations for non-athletes or sedentary individuals (tier 0, [26]), military personnel, athletes with disabilities, pregnant athletes or athletes with clinical disorders. Papers with recommendations on the use of substances prohibited by the World Anti-Doping Agency were also excluded. When authors could not be contacted to retrieve full texts, papers were excluded.

#### Search Strategy and Databases

Electronic databases SCOPUS, PubMed, SPORTDiscus, CINAHL Complete and APA PsycINFO were searched for relevant papers without date restrictions and in the English language only (search conducted 19 May 2023 and repeated 2 August 2024). The search strategy included the following keyword string: (‘athlet*’ OR ‘player*’ OR ‘sport*’) AND (‘body composition’ OR ‘anthro*’ OR ‘weight’ OR ‘mass’ OR ‘fat’ OR ‘muscle’ OR ‘hypertrophy’ OR ‘performance’) AND (‘nutrition’ OR ‘diet’ OR ‘food’) AND (‘guid*’ OR ‘recommend*’ OR ‘position stand’ OR ‘position statement’ OR ‘consensus’) NOT (‘adoles*’ OR ‘youth’ OR ‘junior’). Reference lists of selected papers were manually searched for eligible papers to ensure an exhaustive search. The search strategy of all identified keywords and index terms was adapted for each individual database (see Supplementary Information 2 for example of search strategy used).

### Stage 3: Study Selection

**Fig. 1:**
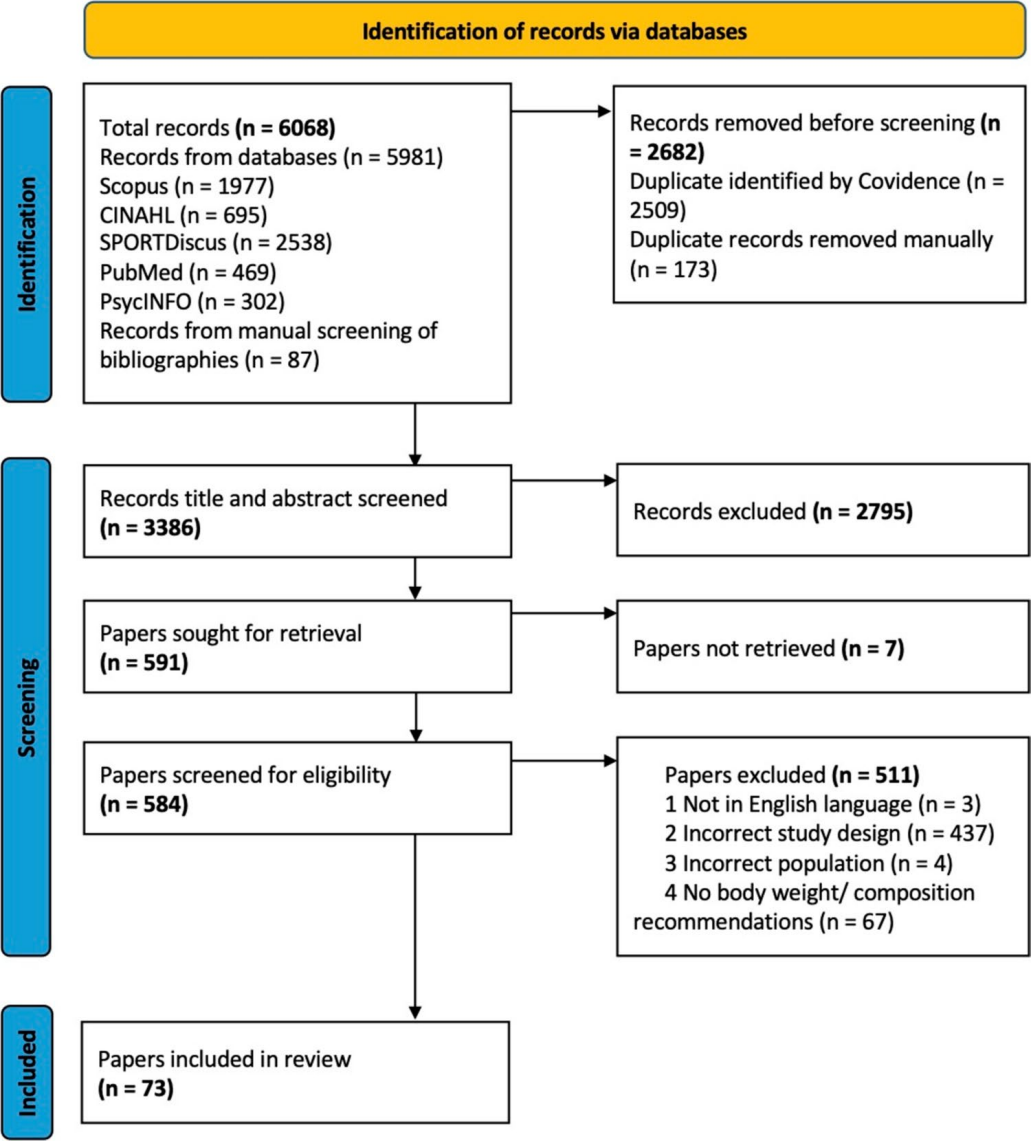
PRISMA-SCR flow diagram for paper inclusion

Search results were uploaded to Covidence systematic review software (Veritas Health Innovation, Melbourne, Australia), and duplicates were removed both automatically and manually. A two-stage screening process was used to select studies: title and abstract, and full text. Initially, title and abstract screening were piloted with 10% of records to ensure reliability between authors. Subsequently, all records were screened against eligibility criteria independently by L.D. and N.C. Full-text publications were then retrieved for the remaining records and screened for inclusion by the same authors. Any conflicts at each stage of the screening process were resolved through discussion between the authors or with S.B. The final full-text publications (*n* = 73) went through the data charting and extraction process. The results of this search and study inclusion process are reported in full in the results section and are presented in a PRISMA-ScR flow diagram (Fig. 1).

### Stage 4: Charting the Data

Covidence systematic review software was used for the extraction and charting process. A data extraction template was developed and reviewed iteratively by the research team, with piloting on five papers by L.D. and N.C. to ensure reliability. Both authors were registered practitioners with the Sport and Exercise Nutrition Register (SENR) and practicing nutritionists in elite sport. Following this, full data extraction was completed by L.D., however, discussions took place with the research team when questions arose about the data charting process. General characteristics (year of publication, authors and country of residence, members of expert group, name of organisation, type of study, aims of study, target population, conflicts and funding information) were extracted. Data related to the development of recommendations and the process of gaining consensus were also extracted. When a paper did not include the consensus process or expert panel members but was an outcome of a wider consensus conference, this information was extracted from the parent protocol paper. The dietary recommendations for increasing and decreasing body mass and/or body composition was extracted.

### Stage 5: Collating, Summarising and Reporting

Since scoping reviews focus on mapping the existing literature rather than producing specific results or outcomes [21], data were summarised and presented descriptively in tables and figures. Paper characteristics and the process of developing the recommendations were outlined together. The level of athlete described was not detailed enough to categorise levels on the basis of the McKay tiered system [26], therefore the phrases used by each paper was kept. Recommendations describing the process of creating body mass and composition goals, including the end targets, rate of change or time of year, were organised separately. Diet and supplement recommendations were categorised on the basis of the objective of either increasing body or fat-free mass or decreasing body or fat mass. Recommendations that did not specify a particular target (i.e. those focussing on ‘optimising’ body composition without clear indication of what change is being made to body mass, fat and fat-free mass) were included in both categories. Guidance that included a combination of changes, for example, increasing fat-free mass at the same time as decreasing fat mass, were also included in both categories. The language used in papers to describe recommendations was preserved regardless of accuracy, for example, synonyms for fat-free mass were kept consistent (e.g. muscle mass or lean mass). Descriptors were only altered to improve conciseness or clarity.

## Results

### Search Results

The full electronic search yielded 6068 records (5385 from original and 596 from repeat search), with 73 papers (4,29–100) identified for inclusion in the review after the removal of duplicates and screening for eligibility. Figure 1 presents a flow diagram detailing the process from study identification.

### Paper Characteristics

**Fig. 2:**
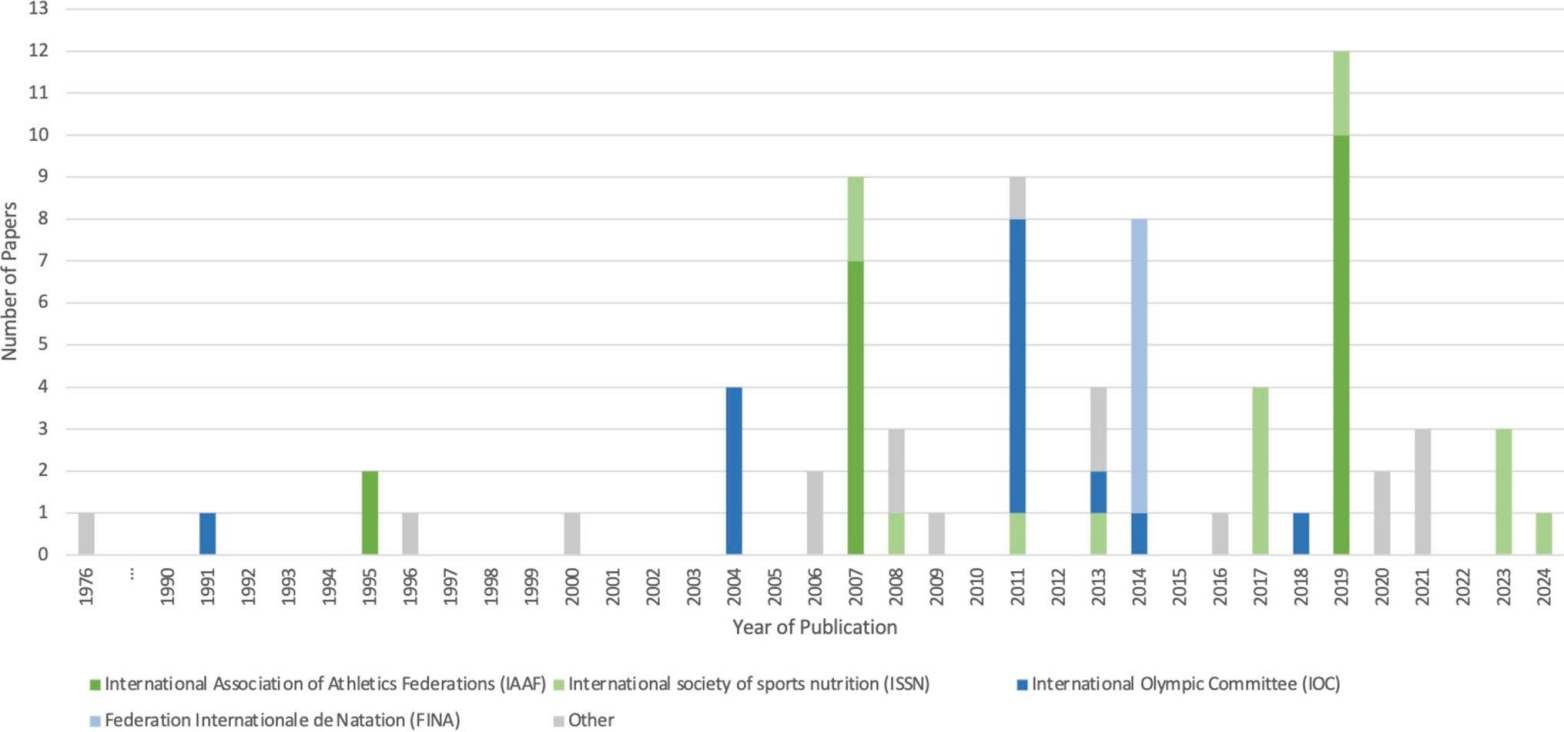
Timeline of publication year for each paper by organisation

Table 1 summarises the characteristics of all papers included in this study: five papers were published in the 1990 s, 20 in the 2000 s, 39 in the 2010 s and nine in the 2020s. A total of 14 organisations endorsed the recommendations, with the majority of papers (*n* = 56) being developed by four international sporting or nutrition organisations: the International Association of Athletics Federation (now World Athletics) (*n* = 19), the International Olympic Committee (*n* = 15), the International Society of Sports Nutrition (*n* = 15) and the Federation Internationale de Nataton (now World Aquatics) (*n* = 7) (Table 1). Most organisations were either international (*n* = 48) or based in the USA (*n* = 21) (Table 1). Figure 2 shows a timeline of the number of papers published each year according to organisation.

**Table 1:** Summary of the characteristics of all included studies, by organisation

Name of affiliated organisation	Author (year) title	Nature of contribution	Stated aim	Is it a series update?	Summary of expert group/number of experts	Summary of consensus process	Evidence grading system used?	Literature review completed?	Sport category/specific sport/level of athlete	Funding type/declared conflicts of interest
Academy of Nutrition and Dietetics, Dietitians of Canada, and The American College of Sports Medicine	Manore et al. (2000) Nutrition and athletic performance [34]	Joint position stand	Review the current scientific data related to the energy needs of athletes, assessment of body composition, strategies for weight change, the nutrient and fluid needs of athletes, special nutrient needs during training, the use of supplements and nutritional ergogenic aids and the nutrition recommendations for vegetarian athletes	Yes [4, 53]	Dietitians and nutritionists from Academy of Nutrition and Dietetics, Dietitians of Canada, and the American College of Sports Medicine/ 17	Not stated	Not stated	Not stated	All sports/ Not applicable/ Not stated	Not stated/ Not stated
	Rodriguez et al. (2009) Nutrition and athletic performance [53]	Joint position stand	Analyse nutrition and performance-specific literature with current scientific data related to energy needs, assessment of body composition, strategies for weight change, nutrient and fluid needs, special nutrient needs during training and competition, the use of supplements and ergogenic aids, nutrition recommendations for vegetarian athletes and the roles and responsibilities of the sports dietitian	Yes [4, 34]	See Manore et al. 2000 above/ 28	Systematic review Used the ADA Evidence Analysis Process and information from the ADA Evidence Analysis Library	Yes – Conclusion Statements are assigned a grade by an expert work group: grade I, II, III, IV, V	Yes – Systematic review – Full search process included	All sports/ Not applicable/ Competitive and recreational	Not stated/ Not stated
	Thomas et al. (2016) Nutrition and athletic performance [4]	Joint position stand	Outline the current energy, nutrient and fluid recommendations for active adults and competitive athletes	Yes [34, 53]	See Manore et al. 2000 above/ 22	ADA Evidence Analysis Process and information from the ADA Evidence Analysis Library	Yes – Conclusion Statements are assigned a grade by an expert work group: grade I, II, III, IV, V	Yes – Systematic review	All sports/ Not applicable/ Competitive and recreational	Not stated/ Not stated
American College of Sports Medicine	American College of Sports Medicine (1976) American College of Sports Medicine position stand on weight loss in wrestlers [29]	Position stand	Not stated	Yes [33, 93]	Three to five experts will be identified by the Pronouncements Committee and chosen to write the paper. Authors will be content experts with an advanced degree pertaining to the subject and have published on the given topic in a peer-reviewed journal in the past 3–5 years. Ideally, at least one of the authors will be mid-career/ Not stated	Research evidence and consensus of expert opinion used. Process: scientific/clinical question, brief synopsis of literature, expert interpretation of literature and key future directions or references	Not stated	Yes – Brief synopsis of literature	Weight category sport/ Wrestling/ High school and college	Not stated/ Not stated
	Oppliger et al. (1996) American College of Sports Medicine position stand: Weight loss in wrestlers [33]	Position stand	To replace the 1976 ACSM position paper,'Weight Loss in Wrestlers'	Yes [29, 93]	Authors and American College of Sports Medicine member reviewed/ 9	See ACSM 1976 above	Not stated	Yes – Brief synopsis of literature	Weight category sport/ Wrestling/ High school and college	Not stated/ Not stated
	Burke et al. (2021) American College of Sports Medicine expert consensus statement on weight loss in weight-category sports [93]	Consensus statement	Recent research has highlighted the specificity and nuances of body mass manipulation practices within each sport and the need for practitioners to support pragmatic strategies for management of body mass around competition to optimize performance whilst safeguarding health. This consensus statement provides a summary of factors that should be considered and replaces the 1996 American College of Sports Medicine Position Stand on Weight Loss in Wrestlers	Yes [29, 33]	Authors/ 5	See ACSM 1976 above	Not stated	Yes – Brief synopsis of literature	Weight category sport/ Combat (e.g. boxing, martial and mixed martial arts [MMA], wrestling), weightlifting, powerlifting, sprint football and rowing. Not horse racing/ Not stated	No financial disclosures/ None
Australian Institute of Sport and National Eating Disorders Collaboration	Wells et al. (2020) The Australian Institute of Sport (AIS) and National Eating Disorders Collaboration (NEDC) position statement on disordered eating in high performance sport [3]	Joint position stand	To guide the clinical management of disordered eating in high performance sport	No	Authors and reviewed by other organisations including National Eating Disorders Collaboration Steering Committee, the Australia and New Zealand Academy for the Eating Disorders Executive Team and the Butterfly Foundation Prevention Services/ 7	Not stated	Not stated	Not stated	All sports/ Not applicable/ High performance	Funded through staff allocation by the Australian Institute of Sport and the National Eating Disorders Collaboration/ None
Fédération Internationale De Football Association	Burke et al. (2006) Energy and carbohydrate for training and recovery [39]	Consensus statement	Review the players'needs for energy and carbohydrate to fuel, recover and optimise the adaptations from these sessions	Yes [119]*	Fédération Internationale de Football Association group/ 36	A group of international experts spent 3 days reviewing the evidence relating to nutrition and soccer. Focus on research undertaken over the last decade	No	Unclear – Reviewed the evidence	Team sport/ Football/ Not stated	Not stated/ Not stated
	Hespel et al. (2006) Dietary supplements for football [40]	Consensus statement	Discuss the relevance of supplement intake in football from a scientific perspective, together with some ethical concerns associated with supplement intake and sports education	No	See Burke et al. 2006 above/ 36	A group of international experts spent 3 days reviewing the evidence relating to nutrition and soccer	No	Unclear – Reviewed the evidence	Team sport/ Football/ Not stated	Not stated/ Not stated
Federation Internationale De Natation	Benardot et al. (2014) Nutritional recommendations for divers [67]	Consensus statement	Not stated	No	Conference with leading sport nutrition scientists and aquatic clinician experts from the around the world/ 4 authors	The sports nutrition scientific evidence for all the aquatic disciplines was reviewed, presented, debated and eventual consensus on evidence-based recommendations was accomplished	No	Unclear – Reviewed scientific evidence	Aquatic sport/ Diving/ Not stated	Financial assistance for conference from Yakult/ None
	Cox et al. (2014) Nutritional recommendations for water polo [68]	Consensus statement	Provide an overview of water polo and highlight the physiological and nutritional demands associated with daily training and water polo match play. The nutritional intake and nutrition-related issues specific to this population are discussed, as well as nutritional interventions likely to enhance the response to daily training and performance during training and match play	No	See Benardot et al. 2014 above/ 3 authors	See Benardot et al. 2014 above	No	Unclear – Reviewed scientific evidence	Aquatic sport/ Water polo/ Not stated	Financial assistance for conference from Yakult/ None
	Derave et al. (2014) Dietary supplements for aquatic sports [69]	Consensus statement	Not stated	No	See Benardot et al. 2014 above/ 2 authors	See Benardot et al. 2014 above	No	Yes – Some process included	Aquatic sport/ Not applicable/ Not stated	Financial assistance for conference from Yakult/ Not stated
	Melin et al. (2014) Disordered eating and eating disorders in aquatic sports [70]	Consensus statement	The aim of this review is to address gaps in knowledge and practice by (a) defining the disordered eating continuum and reviewing the prevalence of disordered eating/eating disorders, (b) summarising the risk factors and consequences that are generally associated with RED-S and finally (c) suggesting strategies for the management and prevention of disordered eating/eating disorders in athletes competing in diving, synchronised swimming and swimming	No	See Benardot et al. 2014 above/ 5 authors	See Benardot et al. 2014 above	No	Unclear – Reviewed scientific evidence	Aquatic sport/ Not applicable/ Not stated	Financial assistance for conference from Yakult/ None
	Mujika et al. (2014) Nutrition and training adaptations in aquatic sports [72]	Consensus statement	Provide a brief overview of long- and short-term training issues relevant for coaches, athletes and support teams to better address the performance requirements of aquatic sports and to address various nutritional strategies that may affect training adaptation	No	See Benardot et al. 2014 above/ 3 authors	See Benardot et al. 2014 above	No	Unclear – Reviewed scientific evidence	Aquatic sport/ Not applicable/ Not stated	Financial assistance for conference from Yakult/ None
	Robertson et al. (2014) Nutritional recommendations for synchronized swimming [73]	Consensus statement	Review the existing sport science literature on nutrition in synchronised swimming	No	See Benardot et al. 2014 above/ 3 authors	See Benardot et al. 2014 above	No	Yes – Intensive literature review	Aquatic sport/ Synchronised swimming/ Not stated	Financial assistance for conference from Yakult/ None
	Shaw et al. (2014) Nutrition for swimming [74]	Consensus statement	Investigate the training and competition characteristics of pool swimming and to discuss nutritional strategies that are important in optimising the outcomes of each component	No	See Benardot et al. 2014 above/ 4 authors	See Benardot et al. 2014 above	No	Unclear – Reviewed scientific evidence	Aquatic sport/ Swimming/ Not stated	Financial assistance for conference from Yakult/ None
International Association of Athletics Federations	Burke (1995) Practical issues in nutrition for athletes [31]	Consensus statement	Review the more common issues of poor nutrition practice and provide general strategies for improved food and fluid intakes	No	Unclear/ Unclear	Proceedings of an international scientific consensus conference	Not stated	Not stated	Athletics – track and field/ Not applicable/ Not stated	Not stated/ Not stated
	Williams (1995) Nutritional ergogenics in athletics [32]	Consensus statement	Review the efficacy of postulated nutritional ergogenic aids regarding biomechanical attributes, primarily body composition, and physiological attributes, primarily energy production	Yes [46, 87]	Unclear/ Unclear	See Burke 1995 above	Not stated	Not stated	Athletics – track and field/ Not applicable/ Not stated	Not stated/ Not stated
	Burke et al. (2007) Nutrition for distance events [42]	Consensus statement	Provide an overview of the major nutritional issues in long-distance running and walking related to optimal physique, training and race day performance	No	International Association of Athletics Federations meeting involved people from a range of professions, including research, sports medicine and clinical nutrition practice, and from different regions around the world, so that a true range of opinions could be represented/ 33	2 days of the consensus conference were spent on an in-depth discussion of the 12 topics. The lead author of each review made a formal presentation of key points, before handing over to co-authors to host a debate	Guidelines for‚ guidelines against and equivocal guidelines	Not stated	Athletics/ Long distance: track and field, road running, cross country, and race-walking/ Competitive and recreational	Parent protocol paper – Financial support for the conference from Powerade/ Not stated
	Houtkooper et al. (2007) Nutrition for throwers, jumpers, and combined events athletes [44]	Consensus statement	Not stated	No	See Burke et al. 2007 above/ 33	See Burke et al. 2007 above	Guidelines for‚ guidelines against and equivocal guidelines	Not stated	Athletics—track and field/ Throwing, jumping, combined events/ Elite	Parent protocol paper – Financial support for the conference from Powerade/ Not stated
	Manore et al. (2007) The female athlete triad: components, nutrition issues, and health consequences [45]	Consensus statement	Review the components of the female athlete triad: energy availability, menstrual status and bone health	No	See Burke et al. 2007 above/ 33	See Burke et al. 2007 above	Guidelines for‚ guidelines against and equivocal guidelines	Not stated	All sports/ Not applicable/ Not stated	Parent protocol paper – Financial support for the conference from Powerade/ Not stated
	Maughan et al. (2007) The use of dietary supplements by athletes [46]	Consensus statement	Review some of the general issues relating to the use of dietary supplements and will look in detail at a few supplements that may have something to offer to some athletes	Yes [32, 87]	See Burke et al. 2007 above/ 33	See Burke et al. 2007 above	Guidelines for‚ guidelines against and equivocal guidelines	Not stated	Athletics – track and field/ Not applicable/ Not stated	Parent protocol paper – Financial support for the conference from Powerade/ Not stated
	O’Connor et al. (2007) Physique & performance for track & field events [47]	Consensus statement	Discuss the relative importance of physique for elite level performance in athletics and evaluate strategies used to achieve loss of weight and body fat	No	See Burke et al. 2007 above/ 33	See Burke et al. 2007 above	Guidelines for‚ guidelines against and equivocal guidelines	Not stated	Athletics – track and field/ Not applicable/ Elite	Parent protocol paper – Financial support for the conference from Powerade/ Not stated
	Stellingwerff et al. (2007) Nutritional strategies to optimize training and racing in middle-distance athletes [48]	Consensus statement	Outline nutrition recommendations during training and racing specific to middle-distance athletes, with an emphasis on the 800-m and 1500-m events	Yes [89]	See Burke et al. 2007 above/ 33	See Burke et al. 2007 above	Guidelines for‚ guidelines against and equivocal guidelines	Not stated	Athletics – track and field/ Middle distance (emphasis on the 800-m and 1500-m events)/ Not stated	Parent protocol paper – Financial support for the conference from Powerade/ Not stated
	Tipton et al. (2007) Nutrition for the sprinter [49]	Consensus statement	Review the role of nutrition for increasing muscle mass and strength, as well as the potential for nutritional choices to influence competition day performance	Yes [88]	See Burke et al. 2007 above/ 33	See Burke et al. 2007 above	Guidelines for‚ guidelines against and equivocal guidelines	Not stated	Athletics – track and field/ Sprinting (from 60 to 400 m, running and hurdles)/ Not stated	Parent protocol paper – Financial support for the conference from Powerade/ Not stated
	Burke et al. (2019) International Association of Athletics Federations consensus statement 2019: Nutrition for athletics [80]	Consensus statement	Summarise the contemporary principles of sports nutrition, identifying strategies that may be used by competitors in athletics to enjoy a long, healthy, and successful career in their chosen event	No	All authors plus expert group from parent protocol paper/ 50	The IAAF has recently commissioned a review of the current status of knowledge, attitudes/cultures, practices and opportunities for sports nutrition to be specifically applied to events in Athletics	Not stated	Not stated	Athletics/ Not applicable/ Elite and recreational	Not stated/ None
	Burke et al. (2019) Contemporary nutrition strategies to optimize performance in distance runners and race walkers [81]	Consensus statement	Review knowledge that has emerged over the past decade on nutrition strategies to support the training and competition goals of distance runners and race walkers, translating race nutrition principles into practical recommendations	No	See Burke et al. 2019 above/ 50	See Burke et al. 2019 above	Not stated	Not stated	Athletics – track and field/ Long distance: track and field, road running, cross country and race-walking/ Not stated	Not stated/ None
	Close et al. (2019) Nutrition for the prevention and treatment of injuries in track and field athletes [82]	Consensus statement	Review nutritional strategies to assist with the most common injuries, that is, skeletal muscle, bone, tendon and ligament, including nutrition to prevent injuries and increase repair, as well as considering the change in energy requirements during the injury period	No	See Burke et al. 2019 above/ 50	See Burke et al. 2019 above	Not stated	Not stated	Athletics – track and field/ Not applicable/ Not stated	Not stated/ None
	Desbrow et al. (2019) Nutrition for special populations: young, female, and masters athletes [83]	Consensus statement	Review aspects of physiology, psychology, training science and sociology to describe our current understanding of the nutrition priorities for these special population athletes	No	See Burke et al. 2019 above/ 50	See Burke et al. 2019 above	Not stated	Not stated	Athletics/ Not applicable/ Not stated	Not stated/ None
	Lis et al. (2019) Dietary practices adopted by track-and-field athletes: gluten-free, low fodmap, vegetarian, and fasting [85]	Consensus statement	Discuss the current state of knowledge, potential implications of select special diets and practical considerations for implementation of these for track-and-field athletes aiming to optimise nutrition for health and performance	No	See Burke et al. 2019 above/ 50	See Burke et al. 2019 above	Not stated	Not stated	Athletics – track and field/ Not applicable/ Not stated	Not stated/ None
	Melin et al. (2019) Energy availability in athletics: health, performance, and physique [86]	Consensus statement	Describe'low energy availability'and potential physiological and psychological consequences in the context of athletics and to provide recommendations regarding prevention, early detection and treatment to achieve safe participation in sport for optimal health and performance	No	See Burke et al. 2019 above/ 50	See Burke et al. 2019 above	Not stated	Not stated	Athletics – track and field/ Not applicable/ Not stated	Not stated/ None
	Peeling et al. (2019) Sports foods and dietary supplements for optimal function and performance enhancement in track-and-field athletes [87]	Consensus statement	Present general considerations for track and field athletes using sports foods and dietary supplements to enhance performance, in addition to exploring the potential therapeutic/prophylactic use of these nutritional aids	Yes [32, 46]	See Burke et al. 2019 above/ 50	See Burke et al. 2019 above	Not stated	Not stated	Athletics – track and field/ Not applicable/ High performance and recreational	Not stated/ Not stated
	Slater et al. (2019) Sprinting... Dietary approaches to optimize training adaptation and performance [88]	Consensus statement	Update the previous International Amateur Athletics Federation consensus on the role of nutrition in elite sprint performance	Yes [49]	See Burke et al. 2019 above/ 50	See Burke et al. 2019 above	Not stated	Not stated	Athletics – track and field/ Sprinting/ High performance and recreational	Not stated/ None
	Stellingwerff et al. (2019) Contemporary nutrition interventions to optimize performance in middle-distance runners [89]	Consensus statement	Provide an evidence-based update since the last International Association of Athletics Federations (IAAF) consensus meeting on contemporary nutrition recommendations to optimise adaptation to training and enhance competition performance in elite middle-distance athletes. This review will focus exclusively on key novel interventions for middle-distance athletes	Yes [48]	See Burke et al. 2019 above/ 50	See Burke et al. 2019 above	Not stated	Not stated	Athletics – track and field/ Middle distance (from 800 to 5000 m)/ Elite	Not stated/ Not stated
	Witard et al. (2019) Dietary protein for training adaptation and body composition manipulation in track and field athletes [90]	Consensus statement	Update the latest evidence-based protein recommendations for training adaptation and promoting, if desired, high-quality weight loss in athletes, with specific application to track and field athletes	No	See Burke et al. 2019 above/ 50	See Burke et al. 2019 above	Not stated	Not stated	Athletics – track and field/ Not applicable/ Not stated	Not stated/ Yes
International Olympic Association	Lemon (1991) Effect of exercise on protein requirements [30]	Consensus statement	To (a) identify the key factors responsible for any increased protein need associated with exercise and (b) provide dietary recommendations for different types of athletes	No	Expert scientists, research scientists and International Olympic Committee delegates from 15 countries and 4 continents/ Unclear	Leading international scientists reviewed the current state of knowledge regarding the role of nutrition in improving sports performance. Scientists, widely recognised as experts in their respective fields, presented reviews of current scientific knowledge in their areas of expertise. For two productive days the manuscripts were individually presented, and open discussions were enjoined by all the participants	Not stated	Not stated	All sports/ Not applicable/ Competitive and recreational	Support from Mars Incorporated for conference/ Not stated
	Loucks (2004) Energy balance and body composition in sports and exercise [35]	Consensus statement	Update, but not replace, the excellent chapter on the state of knowledge about energy balance in sports in the Proceedings of the 1991 International Olympic Committee Consensus Conference on Foods, Nutrition and Sports Performance	No	Mixture of speakers who authored the papers, discussants and other non-experts in nutrition. Included an athlete and EU Anti-doping expert/ 32	This conference followed the format that had worked so well in 1991. Ten authors covered a broad range of topics, and each prepared a manuscript that reviewed the new developments in their allotted area. Two discussants, also experts in the field, were assigned to each of these topics and their remit was to subject the manuscripts to close scrutiny. These manuscripts were circulated to all the conference participants in advance of the meeting. At the conference itself, each author made a short presentation of the key issues, and this was followed by an extended discussion, which was opened by the nominated discussants but to which all the participants made a full contribution	Not stated	Not stated	All sports/ Not applicable/ Not stated	Research grant from US Army Medical Research and Material Command (Defence Women's Health & Military Medical Readiness Research Program) and a grant from the General Clinical Research Branch, Division of Research Resources, National Institutes of Health Parent protocol paper – Assistance from The Coca-Cola Company/ Not stated
	Maughan et al. (2004) Dietary supplements [36]	Consensus statement	Review the various categories of nutritional supplements that are used by athletes and will present evidence for or against the use of selected supplements	No	See Louks 2004 above/ 32	See Louks 2004 above	Not stated	Not stated	All sports/ Not applicable/ Not stated	Parent protocol paper – Assistance from The Coca-Cola Company/ Not stated
	Spriet et al. (2004) Nutritional strategies to influence adaptations to training [37]	Consensus statement	Highlight new nutritional concerns or practices that may influence the adaptation to training	No	See Louks 2004 above/ 32	See Louks 2004 above	Not stated	Not stated	All sports/ Not applicable/ Not stated	Parent protocol paper – Assistance from The Coca-Cola Company/ Not stated
	Tipton et al. (2004) Protein and amino acids for athletes [38]	Consensus statement	Update the literature since 1991 and to critically examine the available information on protein nutrition for athletes	Yes [58]	See Louks 2004 above/ 32	See Louks 2004 above	Not stated	Not stated	All sports/ Not applicable/ Not stated	Parent protocol paper – Assistance from The Coca-Cola Company/ Not stated
	Holway et al. (2011) Sport-specific nutrition: practical strategies for team sports [54]	Consensus statement	Not stated	No	The 2010 conference featured 12 presentations and involved a total of 28 participants, including both research scientists and sports dieticians, from many different countries/ 28	A complete review of the scientific evidence on the relationship between nutrition, performance and health in sport. The scientific papers that formed the basis of that review were presented at the Conference by leading experts in the field and were revised in the light of the discussions that took place The first day of the conference was devoted to a comprehensive review of new developments in the science that underpins the practice of sports nutrition. The second day was devoted to the practical application of this information across a range of sports with different physical and nutritional demands. Each topic was addressed by a single speaker, and two nominated discussants for each session were given the opportunity to open the discussion. Each speaker circulated a manuscript in advance of the conference, allowing all delegates to prepare for a full and open discussion	Not stated	Not stated	Team sport/ Not applicable/ Not stated	Grant for conference from The Coca-Cola Company/ Not stated
	Loucks et al. (2011) Energy availability in athletes [56]	Consensus statement	Update and complement the review of energy balance and body composition in the Proceedings of the 2003 International Olympic Committee Consensus Conference on Sports Nutrition	No	See Holway et al. 2011 above/ 28	See Holway et al. 2011 above	Not stated	Not stated	All sports/ Not applicable/ Not stated	Grant for conference from The Coca-Cola Company/ Not stated
	Meyer et al. (2011) Nutrition for winter sports [57]	Consensus statement	Discuss the winter sport specific environment, altitude and cold, followed by an applied section emphasising the specific nutrition issues faced by winter sport athletes	No	See Holway et al. 2011 above/ 28	See Holway et al. 2011 above	Not stated	Not stated	Winter sports/ Not applicable/ Not stated	Grant for conference from The Coca-Cola Company/ Not stated
	Phillips et al. (2011) Dietary protein for athletes: From requirements to optimum adaptation [58]	Consensus statement	Provide some guidance as to what an athletic ‘optimal’ protein intake might be	Yes [38]	See Holway et al. 2011 above/ 28	See Holway et al. 2011 above	Not stated.	Not stated	All sports/ Not applicable/ Not stated	Grant for conference from The Coca-Cola Company/ Not stated
	Slater et al. (2011) Nutrition guidelines for strength sports: Sprinting, weightlifting, throwing events, and bodybuilding [59]	Expert practice guidelines	Review the nutritional implications of resistance training amongst strength-power athletes, including the sport of bodybuilding	No	See Holway et al. 2011 above/ 28	See Holway et al. 2011 above	Not stated	Not stated	Strength sports/ Sprinting, weightlifting, throwing events and bodybuilding/ Not stated	Grant for conference from The Coca-Cola Company/ Not stated
	Stellingwerff et al. (2011) Nutrition for power sports: middle-distance running, track cycling, rowing, canoeing/kayaking, and swimming [60]	Consensus statement	Outline nutrition recommendations during acute and chronic training and competition, specific to power-based athletes involved in events of 1–10 min duration, including middle-distance running, track cycling, rowing, canoeing/kayaking and swimming. We also highlight body composition considerations and supplements that are relevant to power athletes	No	See Holway et al. 2011 above/ 28	See Holway et al. 2011 above	Not stated	Not stated	Power sports/ Middle-distance running, track cycling, rowing, canoeing/kayaking and swimming/ Not stated	Grant for conference from The Coca-Cola Company/ Not stated
	Sundgot-Borgen et al. (2011) Elite athletes in aesthetic and Olympic weight-class sports and the challenge of body weight and body compositions [61]	Consensus statement	Not stated	No	See Holway et al. 2011 above/ 28	See Holway et al. 2011 above	Not stated	Not stated	Olympic weight category and aesthetic sports/ Not applicable/ Elite	Grant for conference from The Coca-Cola Company/ Not stated
	Sundgot-Borgen et al. (2013) How to minimise the health risks to athletes who compete in weight-sensitive sports review and position statement on behalf of the ad hoc research working group on body composition, health and performance, under the auspices of the IOC medical commission [64]	Position stand	Review the current knowledge related to minimising the risks associated with extreme weight control and eating disorders in elite athletes	No	Authors/ 7	Used existing literature and best practice to suggest guidelines for minimising risk in weight-sensitive sports	Not stated	Not stated	Weight-sensitive sports/ (1) Gravitational sports: long-distance running, cross-country skiing, road and mountain bike cycling, ski jumping and jumping in athletics. (2)Weight-class sports: wrestling, judo, boxing, taekwondo, weightlifting and lightweight rowing. (3) Aesthetically judged sports: rhythmic and artistic gymnastics, figure skating, diving and synchronised swimming/ Elite	Meetings of the Ad Hoc Research Working Group on Body Composition, Health and Performance were financed by the International Olympic Committee/ None
	Mountjoy et al. (2014) The IOC consensus statement: Beyond the female athlete triad: relative energy deficiency in sport (RED-S) [71]	Consensus statement	Update and replace the 2005 International Olympic Committee (IOC) Consensus Statement and the IOC Position Stand on the Female Athlete Triad and provide guidelines to the athlete health support team to guide risk assessment, treatment and return-to-play decisions for affected athletes	Yes [124]*	Authors, including author R.B. who was the Director International Olympic Committee Medical & Scientific Department, and author A.L. who was the Chairman of International Olympic Committee Medical Commission/ 11	On the basis of scientific evidence published in the intervening period, this Consensus Statement serves to update and replace these documents and provide guidelines to the athlete health support team to guide risk assessment, treatment and return-to-play decisions for affected athletes	Not stated	Not stated	All sports/ Not applicable/ Not stated	Funding for consensus meeting by International Olympic Committee/ None
	Maughan et al. (2018) International Olympic Committee consensus statement: Dietary supplements and the high-performance athlete [79]	Consensus statement	Summarise the issues faced by high-performance athletes and their support team (coach, trainer, nutritionist, physician) when considering the use of supplements, with the goal of providing information to assist them to make informed decisions	No	Authors/ 25	Not stated	Not stated	Not stated	All sports/ Not applicable/ High performance	Not stated/ Not stated
International Society of Sports Nutrition	Buford et al. [41] International Society of Sports Nutrition position stand: Creatine supplementation and exercise [41]	Position stand	Determine the present state of knowledge concerning creatine supplementation, so that reasonable guidelines may be established and unfounded fears diminished in regard to its use	Yes [78]	Authors/ 10	The following literature review has been prepared by the authors in support of the aforementioned position statement	Not stated	Yes	All sports/ Not applicable/ Not stated	Not stated/ Not stated
	Campbell et al. (2007) International Society of Sports Nutrition position stand: Protein and exercise [43]	Position stand	Provide a position stand on the intake of protein for healthy, exercising individuals	Yes [76]	Authors/ 9	Not stated	Not stated	Not stated	All sports/ Not applicable/ Healthy, exercising individuals	Not stated/ None
	Kerksick et al. (2008) International Society of Sports Nutrition position stand: Nutrient timing [51]	Position stand	Highlight, summarise and assess the current scientific literature, and make scientific recommendations surrounding the timed ingestion of carbohydrates, protein and fat	Yes [77]	Authors/ 12	Not stated	Not stated	Not stated	All sports/ Not applicable/ Not stated	Not stated/ Not stated
	La Bounty et al. (2011) International Society of Sports Nutrition position stand: Meal frequency [55]	Position stand	Discuss the various research findings in which meal/eating frequency has been an independent variable in human studies that assess body composition, various health markers, thermic effect of food, energy expenditure, nitrogen retention and satiety	No	Authors/ 12	Not stated	Not stated	Not stated	All sports/ Not applicable/ Competitive and recreational	Not stated/ None
	Wilson et al. (2013) International Society of Sports Nutrition position stand: Beta-hydroxy-beta-methyl butyrate (HMB) [66]	Position stand	Critically analyse the existing literature on HMB supplementation and provide careful recommendations on how to optimise its effects on body composition, strength, power, and aerobic performance across varying levels of age, sex and training status	No	Authors/ 15	A critical analysis of the literature on the use of beta-hydroxy-beta-methyl butyrate (HMB) as a nutritional supplement	Not stated	Unclear – Critical analysis of literature	All sports/ Not applicable/ Varying levels of training status	Not stated/ Yes
	Aragon et al. (2017) International Society of Sports Nutrition position stand: Diets and body composition [75]	Position stand	Provide clarity on the effects of various diets on body composition	No	Authors/ 17	A critical analysis of the literature regarding the effects of diet types (macronutrient composition; eating styles) and their influence on body composition	Not stated	Unclear – Critical analysis of literature	All sports/ Not applicable/ Competitive and recreational	No funding received/ Yes
	Jäger et al. (2017) International Society of Sports Nutrition position stand: Protein and exercise [76]	Position stand	Provide new information and address the most important dietary protein categories that affect physically active individuals across domains such as exercise performance, body composition, protein timing, recommended intakes, protein sources and quality, and the preparation methods of various proteins	Yes [43]	Authors plus 2 others to review/ 24	Not stated	Not stated	Not stated	All sports/ Not applicable/ Competitive and recreational	No funding provided/ Yes
	Kerksick et al. (2017) International Society of Sports Nutrition position stand: Nutrient timing [77]	Position stand	Refine recommendations made related to the timed consumption of carbohydrates and protein and how this can potentially affect the adaptive response to exercise	Yes [51]	Authors/ 19	Not stated	Not stated	Not stated	All sports/ Not applicable/ Competitive and recreational (in particular highly trained individuals)	Not stated/ Yes
	Kreider et al. (2017) International Society of Sports Nutrition position stand: Safety and efficacy of creatine supplementation in exercise, sport, and medicine [78]	Position stand	Update to the current literature regarding the role and safety of creatine supplementation in exercise, sport, and medicine and to update the position stand of International Society of Sports Nutrition related to creatine supplementation	Yes [41]	Authors/ 10	Not stated	Not stated	Not stated	All sports/ Not applicable/ Competitive and recreational	Support to prepare manuscript from Council for Responsible Nutrition/ Yes
	Jäger et al. (2019) International Society of Sports Nutrition position stand: Probiotics [84]	Position stand	Provide an objective and critical review of the mechanisms and use of probiotic supplementation to optimise the health, performance and recovery of athletes	No	Authors/ 25	Review of the current available literature	Not stated	Not stated	All sports/ Not applicable/ Competitive and recreational	No renumeration received/ Yes
	Tiller et al. (2019) International Society of Sports Nutrition position stand: Nutritional considerations for single-stage ultra-marathon training and racing [84]	Position stand	Provide a Position Stand on the nutritional considerations of ultra-marathon training and racing to inform best-practice of athletes, coaches, medics, support staff and race organisers	No	Authors/ 25	Literature review. We have graded the strength of our evidence statements according to the system employed by the National Heart, Lung, and Blood Institute, which we have adapted to incorporate a fourth level pertinent to case-reports	Yes – Grading system and evidence strategies A, B, C, D	Yes – Some process included	Athletics/ Long distance – Single stage ultra-marathon up to and including 152 miles (245 km)/ Not stated	No funding received/ None
	Ferrando et al. (2023) International Society of Sports Nutrition position stand: Essential amino acid supplementation on skeletal muscle and performance [95]	Position Stand	Presents the position of the International Society of Sports Nutrition (ISSN) on the effect of dietary supplementation with free-form essential amino acids on muscle protein synthesis, muscle mass and quality and physical performance	No	Authors/ 22	ISSN position stands are invited papers the ISSN editors and Research Council identify as topics of interest to our readers that need position stands to provide guidance to readers and the profession. Editors and/or the Research Council identify a lead author or team of authors to perform a comprehensive literature review The draft is then sent to leading scholars for review and comment. The paper is then revised as a consensus statement and reviewed and approved by the Research Council and Editors as the official position of the ISSN	Not stated	Yes – Comprehensive literature review	All sports/ Not applicable/ Not stated	Research reported in this publication was supported by the National Center For Advancing Translational Sciences of the National Institutes of Health under award number (TL1 TR003109 and UL1 TR003107)/ Yes
	Lowery et al. (2023) International Society of Sports Nutrition position stand: Coffee and sports performance [96]	Position Stand	Review the complexity of coffee, its role in sports nutrition, and how it interacts with exercise	No	Authors/ 14	ISSN position stands are invited papers of topics the ISSN Editors and Research Council identify as topics of interest to our readers who need Position Stands to provide guidance to readers and the profession. Editors and/or the research committee identify a lead author or team of authors to perform a comprehensive literature review A review of the scientific literature was conducted related to the physiological effects and nutritional aspects of coffee. This was accomplished by conducting keyword searches related to coffee using the National Institutes for Health National Library of Medicine PubMed.gov search engine The draft is then sent to leading scholars for review and comment. The paper is then revised as a consensus statement and reviewed and approved by the Research Committee and Editors as the official position of the ISSN	Not stated	Yes – Comprehensive literature review, details included	All sports/ Not applicable/ Not stated	No funding received/ Yes
	Sims et al. (2023) International Society of Sports Nutrition position stand: Nutritional concerns of the female athlete [97]	Position Stand	Review the research and provide practical evidence-based female-specific recommendations for sport nutrition	No	Authors/ 19	The International Society of Sports Nutrition (ISSN) position stands are invited papers on topics the Journal of the ISSN (JISSN) Editors and Research Committee identifies as being of interest to JISSN readers. The process consists of editors and/or the ISSN Research Committee identifying a lead author or team of authors to perform a comprehensive literature review Specifically, for this Female Athlete Position Stand, the scientific design of the studies was scrutinised for scientific validity as part of the inclusion criteria. After the authors develop the content of the position stand, the draft is sent to leading scholars in the field for a detailed review. Following a critical review by the scholars, the paper was revised by a team of authors, approved by the ISSN Research Committee and JISSN Editors and published as a consensus statement and the official position of the ISSN on the topic	Unclear – Specifically, for this Female Athlete Position Stand, the scientific design of the studies was scrutinised for scientific validity as part of the inclusion criteria	Yes – Comprehensive literature review	All sports/ Not applicable/ Not stated	No funding received/ None
	Leaf et al. (2024) International Society of Sports Nutrition position stand: Ketogenic diets [98]	Position Stand	Discuss the impact of ketogenic diets on athletic performance, muscular strength, resistance training adaptations, and body composition	No	Authors/ 18	ISSN position stands are invited reviews of topics the Journal of the ISSN (JISSN) editors and Research Committee identify as being of interest to the sports nutrition community. JISSN Editors and/or the Research Committee identify a lead author or team of authors to perform a comprehensive literature review A comprehensive literature search was performed using the Medline database of the US National Library of Medicine of the National Institutes of Health (PubMed). To be eligible for inclusion and discussion, studies had to be controlled trials comparing a ketogenic diet – defined as containing < 50 g of carbohydrate per day or resulting in blood ketone values ≥ 0.5 mM or resulting in the presence of urinary ketones – to a non-ketogenic control diet in adults undergoing an exercise regimen The draft is then sent to leading scholars for review and comment. The paper is then revised as a consensus statement and reviewed and approved by the Research Committee and JISSN Editors as the official position of the ISSN	Not stated	Yes – Comprehensive literature review	All sports/ Not applicable/ Not stated	No funding received/ Unclear
National Athletic Trainers'Association	Bonci et al. (2008) National Athletic Trainers'Association position statement: Preventing, detecting, and managing disordered eating in athletes [50]	Position stand	Provide recommendations to better prepare certified athletic trainers, other healthcare providers, sports management personnel and coaches for the challenges of understanding and working with athletes who present with disordered eating or who may be at risk	No	National Athletic Trainers association members/ 12	Scientifically based, peer-reviewed research with a team of authors who are experts on the subject	Not stated	Not stated	All sports/ Not applicable/ Not stated	Not stated/ Not stated
	Turocy et al. (2011) National Athletic Trainers'Association position statement: Safe weight loss and maintenance practices in sport and exercise [62]	Position stand	Identify safe methods by which goal weight can be determined and maintained and to discuss unsafe weight management practices and the effects of those practices on performance and overall health	No	See Bonci et al. 2008 above/ 14	Scientifically based, peer-reviewed research with a team of authors who are experts on the subject Used the current research and literature in the area. The recommendations are categorised using the Strength of Recommendation Taxonomy criterion scale proposed by the American Academy of Family Physicians based on the level of scientific data found in the literature – A, B, C	Yes – SORT Criterion scale based on the level of scientific data found in the literature – A, B, C	Not stated	All sports/ Not applicable/ Competitive and recreational	Not stated/ Not stated
	Buell et al. (2013) National Athletic Trainers'Association position statement: Evaluation of dietary supplements for performance nutrition [63]	Position stand	Help athletic trainers promote a ‘food-first’ philosophy to support health and performance, understand federal and sport governing body rules and regulations regarding dietary supplements and banned substances and become familiar with reliable resources for evaluating the safety, purity and efficacy of dietary supplements	No	See Bonci et al. 2008 above/ 14	Scientifically based, peer-reviewed research with a team of authors who are experts on the subject To formalise the position statement objectives into recommendations, we used (where appropriate) evidence-based review and the Strength of Recommendation Taxonomy criterion scale Evaluation of the literature associated with performance nutrition and dietary supplements	Yes – SORT Criterion scale based on the level of scientific data found in the literature – A, B, C	Not stated	All sports/ Not applicable/ Not stated	Not stated/ Not stated
New Zealand Dietetic Association	New Zealand Dietetics Association (2008) Nutrition for Exercise and sport in New Zealand [52]	Position stand	Provide the stand of the New Zealand Dietetic Association on nutrition advice for athletes to follow in everyday training and at times of competition	No	Authors and 2 other dietitians and 1 external/ 7	Not stated	Not stated	Not stated	All sports/ Not applicable/ Elite, recreational sports and exercise	Not stated/ Not stated
Portuguese Football Federation	Abreu et al. (2021) Portuguese Football Federation Consensus statement 2020: Nutrition and performance in football [92]	Consensus statement	Discuss and outline current practices and find consensus in the practical application of nutrition-related strategies to promote health and improve performance for football players	No	18 nutritionists: male football, Portugal only 11 other experts: nutritionists, sports scientists and team physicians working with elite football teams and academia/ 29	Established a network of nutritionists from elite football clubs in Portugal, aiming to discuss and outline current practices and find consensus in the practical application of nutrition-related strategies to promote health and improve performance for football players. Consensus meeting was organised and 17 experts attended. The participants agreed on focussing the discussion on nutritional considerations for adult, male, elite players for the current consensus	Not stated	Not stated	Team sport/ Football/ Elite	No funding received to produce this manuscript/ Yes
Team Physicians Group Includes the Following Organisations: The American Academy of Family Physicians, The American Academy of Orthopaedic Surgeons, The American College of Sports Medicine, The American Medical Society for Sports Medicine, The American Orthopaedic Society for Sports Medicine, and The American Osteopathic Academy of Sports Medicine	Team Physicians (2013) Selected issues for nutrition and the athlete: a team physician consensus statement [65]	Consensus statement	Help the team physician understand selected nutrition issues to advise athletes on issues related to health and optimal performance	No	Doctors only/ 10	This statement was developed by a collaboration of six major professional associations concerned about clinical sports medicine issues; they have committed to forming an ongoing project-based alliance to bring together sports medicine organisations to best serve active people and athletes	Not stated	Not stated	All sports/ Not applicable/ Not stated	Not stated/ Not stated
Union Of European Football Associations	Collins et al. (2021) Union of European Football Associations (UEFA) expert group statement on nutrition in elite football. Current evidence to inform practical recommendations and guide future research [94]	Consensus statement	Provide a narrative synthesis of the current evidence relating to various topics in elite football nutrition	No	Expert group members (*n* = 31 in total) included basic and applied researchers (*n* = 6) and field-based practitioners (*n* = 5); the majority (*n* = 14) had a background of both research and field-based practice and six were Union of European Football Associations Medical Committee members/ 31	The Union of European Football Associations (UEFA) has gathered experts in applied sports nutrition research as well as practitioners working with elite football clubs and national associations/federations to issue an expert statement on a range of topics relevant to elite football nutrition. The expert group provide a narrative synthesis of the scientific background relating to these topics on the basis of their knowledge and experience of the scientific research literature, as well as practical experience of applying knowledge within an elite sports setting. Purpose was to update the knowledge and research about nutrition in elite football The expert statement process was created by a steering committee that identified the topics to be included and compiled a list of research and field-based experts. First drafts of each section were collated by the steering committee to form the basis of the first full draft. This was circulated to all expert group members: the applied researchers focussed on the narrative synthesis of the scientific research literature and the practitioners on the ecological validity in the football setting. Comments were collated and changes made before further review by the expert group. This process continued until agreement was reached on the specific sections and recommendations included	Not stated	Unclear – narrative synthesis of scientific background	Team sport/ Football/ Elite	No funding received/ Yes
Working Group Sport Nutrition of The German Nutrition Society	König et al. (2020) Position of the working group sports nutrition of the German Nutrition Society (DGE): protein intake in sports [91]	Position stand	Describe the protein requirements of athletes, specifically the topics of types of proteins, increase in muscle mass and strength, regeneration and timing of protein intake	No	Authors/ 13	Not stated	Not stated	Not stated	All sports/ Not applicable/ Not stated	Not stated/ None

*Reference not included in this review

The review includes three types of recommendation papers: 45 consensus statements, 27 position stands, and one expert practice guideline (Table 1). Of the 73 papers, 23 were part of a series of updated recommendations (Table 1). In total, there were 328 authors, with the most frequent contributors being Professor Jose Antonio (USA) (*n* = 15), Professor Louise M. Burke (Australia) (*n* = 15), Professor Bill I. Campbell (USA) (*n* = 14) and Professor Richard B. Kreider (USA) (*n* = 14) (Table 1).

**Fig. 3:**
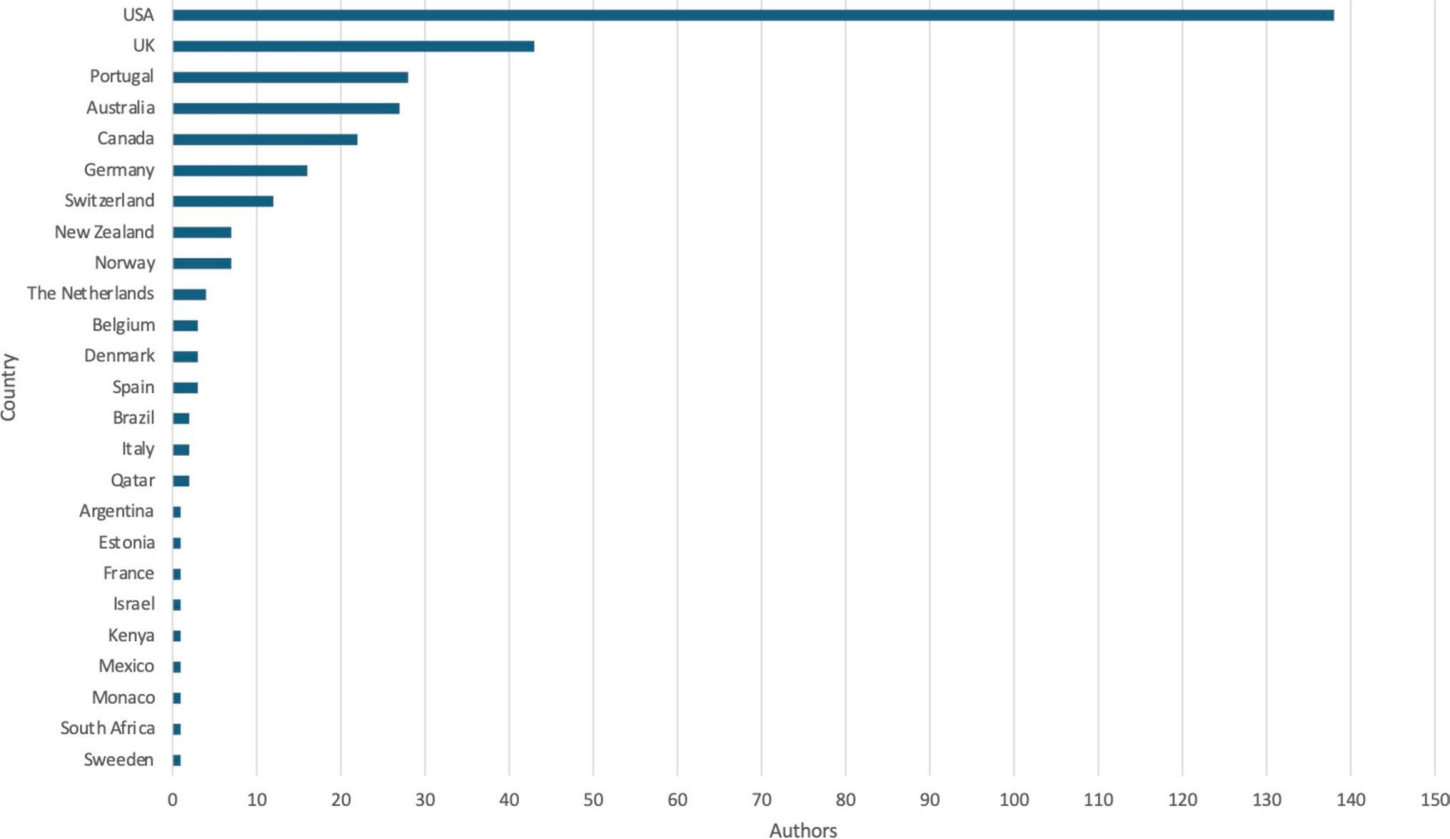
Country of origin of every author

Figure 3 illustrates the global distribution of all authors, with the most common countries represented being the USA (*n* = 138), the United Kingdom (*n* = 43), Portugal (*n* = 28), Australia (*n* = 27), Canada (*n* = 22), Germany (*n* = 16) and Switzerland (*n* = 12). Most papers involved 30–50 experts (*n* = 24) (Table 1). A multidisciplinary team (MDT) of experts, including two or more disciplines, was involved in 35 papers, whilst seven papers involved experts from one discipline alone, mostly dietitians or nutritionists. The professional background of experts was unclear in 31 papers. Four papers from the same conference included one athlete in their MDT of experts when developing recommendations.

Expert conferences were used to create consensus, communicated via 30 papers (Table 1). The use of an evidence-grading system and literature review was not consistently applied; 60 papers did not clearly state the use of an evidence-grading system and 50 papers did not state if they completed a literature review (Table 1). A similar number of papers focussed on general athlete recommendations (*n* = 34) versus sport specific (*n* = 39) (Table 1). Amongst those that did specify a sport, the most common categories included: athletics (*n* = 19), aquatic sports (*n* = 7), team sports (*n* = 5) and weight category sports (*n* = 4).

A total of 45 papers did not state the level of athlete targeted by the recommendations (Table 1). The two most frequent descriptors of level of athlete included ‘competitive and recreational’ (*n* = 11) and ‘elite’ (*n* = 7). A total of 67 papers did not identify the sex of the target population, and of those that did, two were for female and male athletes, two were for female athletes only, one for male athletes only and one for male athletes with a female-athlete-only section. A total of 40 papers declared whether they had funding, including 30 papers declaring funding and 10 declaring no funding (Table 1), and 33 papers declared whether there were conflicts of interest, with 11 declaring conflicts and 22 declaring no conflicts (Table 1).

### Recommendations on Goal-Setting of Body Mass and/or Composition

Of 73 papers, 40 provide recommendations on athlete body mass or composition outcome targets, the rate of achieving these outcomes or the timing of such changes [3, 4, 29, 31, 33–35, 38, 39, 42, 44, 47–50, 52–54, 57–62, 64, 65, 68, 70, 72, 74, 75, 80, 86, 88–90, 92–94, 97]. Table 2 synthesises the key outcome recommendations, only including those recommended by three or more papers.

**Table 2:** Synthesis of goal-setting recommendations for target setting, rates of change and timing when altering athlete body mass and composition

Recommendation	References
*Target*	
Choose body mass and composition targets that are individual to each athlete	[4, 34, 39, 42, 53, 61, 70, 74, 64, 62, 92, 94]
Consider the requirements of the sport/discipline when setting body mass and composition targets	[4, 34, 35, 53, 60, 65, 74, 62]
Avoid body fat level below 5% in male athletes and below 12% in female athletes	[34, 52, 53, 33, 61, 64, 62]
Consider genetics/heredity factors when setting body mass and composition targets	[4, 34, 42, 53, 90]
Choose body mass and composition targets that are realistic	[4, 34, 42, 53, 65]
Choose body mass and composition targets that are healthy and support performance	[34, 53, 62, 80, 93]
Consider age when setting body mass and composition targets	[4, 34, 53, 65]
Aim to maintain fat-free mass and decrease fat mass to decrease body mass	[4, 65, 72, 80]
Consider sex of the athlete when setting body mass and composition targets	[4, 34, 53]
Consider playing position of the athlete when setting body mass and composition targets	[39, 68, 94]
Consider that optimal for an athlete may be above the minimum safe body fat level when setting body mass and composition targets	[34, 53, 93]
Avoid an unnaturally low/too little/below biological default body fat level	[31, 34, 42]
Choose a target range for body mass and composition and not a specific or absolute target	[4, 34, 74]
Avoid increasing mass that does not increase power and the power:mass ratio (i.e. there is an upper limit of fat-free mass)	[49, 80, 88]
Aim to increase power:mass ratio	[90, 80, 97]
Use reference data for body mass and composition that is sport or event specific	[47, 54, 62]
*Rate*	
Aim for a gradual/slow decrease in body mass	[4, 34, 35, 39, 47, 48, 61, 62, 75]
Aim to decrease body/fat mass at a rate of 0.5–1.0 kg/week or 1–2 lb/week	[31, 34, 52, 61, 90, 64, 62]
*Timing*	
Aim to change body mass early in the season or before the competitive season starts	[4, 34, 44, 52, 53, 54, 60, 86, 88]
Aim to change body mass in the offseason	[34, 39, 44, 53, 61, 65, 64, 92]
Aim to make body composition changes in the pre-season/general preparation phase	[39, 48, 54, 60, 62, 72, 92, 94]
Aim for periodised changes in body composition across a season	[89, 74, 80]

The importance of identifying body mass and composition outcome targets that are individual to each athlete was clearly advised by 12 papers (Table 2). Considering the requirements of the sport (*n* = 8), genetics (*n* = 5), age (*n* = 4), sex (*n* = 3) and playing position (*n* = 3) was also acknowledged when setting body mass and body composition outcome goals (Table 2). Avoiding a body fat level below 5% for men and 12% for women was recommended by seven papers, along with choosing realistic targets (*n* = 5) that support health and performance (*n* = 5) (Table 2). Three papers advised to consider that optimal body fat targets may be above the minimum safe levels (Table 2). Three papers recommended setting goals on the basis of increasing power:mass ratio, three papers advised avoiding increasing mass that does not increase power and three papers advised a focus on decreasing fat mass and maintaining muscle mass to decrease body mass (Table 2).

A gradual decrease in body mass (*n* = 9), at a rate of 0.5 to 1 kg per week (*n* = 7), was the most common advice provided on the rate of change when recommendations were provided (Table 2). The timing of any change in body mass or composition was considered, with emphasis placed on making such changes before the competition season (*n* = 9), with the off-season (*n* = 8) or pre-season being ideal (*n* = 8) (Table 2). It was also recommended to periodise changes in body composition across the season (*n* = 3).

### Diet and Supplement Recommendations to Manipulate Body Mass or Composition

**Fig. 4:**
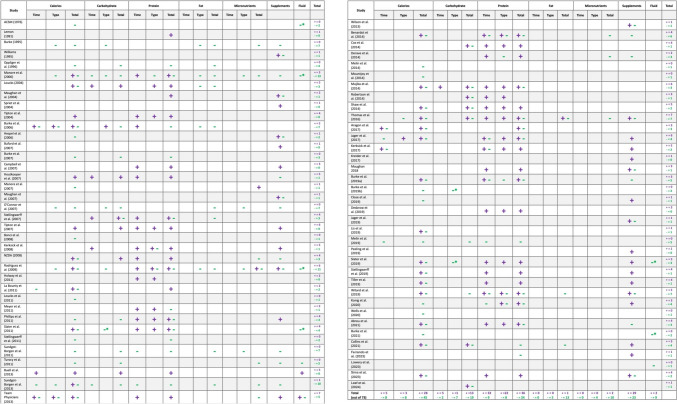
Overview of the dietary recommendations included in all papers

An overview of the key components of dietary recommendations in each paper, including calories, macronutrients, micronutrients, supplements and fluid, was summarised in Fig. 4.

#### Diet and Supplement Recommendations to Increase Body Mass or Fat-Free Mass

A total of 55 papers included recommendations to increase body or fat-free mass (Fig. 4) [4, 30, 32, 34–41, 43–46, 48, 49, 51–55, 57–59, 63–69, 72–80, 82–85, 87–92, 94, 95, 97, 98]. Guidance on calorie (*n* = 28), protein (*n* = 40) or supplement intakes (*n* = 29) were most common (Fig. 4). Calorie recommendations were often vague, advising ‘adequate’, ‘enough’, ‘sufficient’ or ‘appropriate’ intakes (supplementary information 3). A total of 11 papers recommended increasing energy intake to increase body or fat-free mass, with three quantifying this increase as 500–1000 kcal/day, and four emphasising individual energy requirements (e.g. different needs of trained versus untrained athletes).

Protein recommendations were the most common and detailed nutrient recommendations provided, addressing daily intake patterns, intake around training, protein types, total daily intake and portion sizes (Fig. 4). Targets for daily protein intake varied widely, with 17 different ranges between 1.2 and 2.5 g/kg body mass/day (*n* = 20) (supplementary information 3). Seven earlier papers recommended absolute portions of 15–40 g protein or 6–40 g amino acids, whilst papers from 2014 moved to relative portions (*n* = 10) (0.25–0.50 g/kg body mass/meal). Protein distribution recommendations varied, with optimal patterns ranging from 3 to 6 feeds per day (*n* = 7) to intervals of 3–5 h (*n* = 4). A total of 29 papers recommended protein intake around training, especially post-training. Beyond training, six papers recommended protein intake before bed, and 22 discussed the type of protein, advocating for high-quality or biological value proteins, intact or whole food proteins and high leucine-based proteins.

Protein or amino acid supplements were most commonly recommended, featuring in 19 papers (supplementary information 3); nine papers recommended protein supplements to increase body or fat-free mass in certain circumstances (e.g. for convenience), particularly whey protein (*n* = 7). Creatine supplementation was recommended in 12 papers to increase body or fat-free mass, with three reporting potential benefits and two recommendations prior to 2004 advising against use due to insufficient evidence (supplementary Information 3). In addition, 11 papers recommended an initial loading dose of 15–20 g/day in four equal doses, followed by a maintenance dose of 1–5 g/day. Only three papers before 2007 recommended a creatine wash-out period of 4–10 weeks.

A total of 15 papers included carbohydrate recommendations to increase body or fat-free mass (Fig. 4). Like protein, recommendations were again vague, and included consuming ‘sufficient’, ‘adequate’ or ‘enough’ carbohydrates to avoid protein oxidation and adapting intakes on the basis of body composition goals. Four papers advised on carbohydrate intake around training and five separate daily carbohydrate targets were recommended in the range of 3–12 g/kg body mass/day (supplementary information 3). Only one paper advised avoiding excessive intakes of fibre-rich foods, as this may reduce appetite and impair total food intake, and one paper recommended against the use of a ketogenic diet for increasing fat-free mass.

Very few papers gave recommendations regarding fat (*n* = 1), micronutrient (*n* = 2) or fluid (*n* = 2) intake to support increases in body or fat-free mass (Fig. 4). Advice included individualising fat intake on the basis of body composition goals, assessing the diet for adequate intake of zinc and B vitamins, considering greater intakes of micronutrients and ensuring sufficient or proper hydration around training (supplementary information 3).

#### Diet and Supplement Recommendations to Decrease Body Mass or Fat Mass

A total of 60 papers included recommendations on decreasing body or fat mass (Fig. 4) [4, 29, 31–36, 39, 40, 42, 44–48, 50–53, 55–62, 64–77, 79–82, 84–86, 88, 3, 89–98]. The focus was primarily on calorie (*n* = 45), carbohydrate (*n* = 24), protein (*n* = 27) and supplement (*n* = 23) intakes (Fig. 4). The most common recommendation was total calorie intake, with 43 papers specifying either a minimum daily calorie range, a minimum energy availability, or a target deficit (supplementary Information 4). The minimum calorie range was different across each paper, but the minimum energy availability was consistently set at 30 kcal/kg fat-free mass/day in 12 out of 13 papers, with only one paper recommending a lower limit of 20–25 kcal/kg fat-free mass/day for body mass loss in male athletes. The recommended calorie deficit ranged from 250 to 1000 calories per day. Additional advice included choosing low-energy density, high-nutrient density foods and distributing calorie intake throughout the day. Only two papers advise not misusing alcohol due to the link with unwise eating habits and negative impact on managing body composition (supplementary Information 4).

Carbohydrate recommendations varied the most of all nutrients due to the unique requirements of different sports but generally advised periodising or adapting intake to support reduced energy intake, with daily carbohydrate recommendations ranging from 3 to 10 g/kg body mass (supplementary Information 4). Six papers highlighted the lower end of the range (3–6 g/kg body mass/day) for decreasing body or fat mass. Increasing nutrient-dense, high-fibre foods and wholegrains was recommended by three papers, whilst two recommended strategic use of low-fibre diets in the days leading up to competition for acute decreases in body mass. One paper advised to consider the use of a ketogenic diet to decrease body mass but warned about the likelihood of decreasing both fat mass and fat-free mass.

In total, 15 different ranges for daily protein intake were recommended by 18 papers (Supplementary Information 4). Before 2007, the range was 1–1.7 g/kg body mass (*n* = 3), between 2007 and 2019 it increased to 1.4–3.1 g/kg body mass (*n* = 9), whereas after 2019, all six papers consistently recommended a narrower range of 1.6–2.4 g/kg body mass daily. Recommendations for protein portion sizes included three papers suggesting in the range of 20–40 g, one recommending above 40 g and three recommending 0.4–0.5 g/kg body mass/meal. Spreading protein intake throughout the day in 3–5 meals or every 3–4 h was also advised (*n* = 5), particularly around training (*n* = 5). High-quality, whole-food protein sources, especially low-fat dairy and meat were commonly recommended.

Supplement guidelines suggested short-term use of a multi-vitamin and mineral supplement (*n* = 9), and protein supplements as a convenient, low-calorie source to retain fat-free mass during energy deficits (*n* = 4) (supplementary Information 4). Supplements specifically for decreasing fat mass were not recommended by three papers and supplements that posed the risk of an anti-doping rule violation were also discouraged (*n* = 3). In addition, 12 different supplements including boron, carnitine, chromium and probiotics were not recommended for decreasing body mass (*n* = 5).

Few papers included recommendations on fat (*n* = 13), micronutrient (*n* = 12) and fluid (*n* = 9) intakes (Fig. 4). Seven different recommendations were provided for total fat intake ranging from 15 to 30% of energy intakes, and three papers advised against excessive restriction below 15% (supplementary Information 4). Some practical dietary recommendations were also included. For example, switching from high-fat to low-fat foods, reducing saturated fat intakes and reducing intakes of fats and oils during cooking.

Eight papers advise to consider micronutrient and antioxidant intakes for athletes on restrictive diets as they are at high risk of deficiency (supplementary Information 4). Aiming for the recommended daily amount, dietary reference intake or adequate intake was recommended by three papers and two recommended consuming five servings of fruits and vegetables daily to ensure sufficient micronutrient intakes. Four papers advised to consider calcium requirements and including low-fat dairy foods to support calcium intakes.

Two papers advised appropriate intakes of fluid to remain hydrated, and one advised against the use of coffee for altering body composition (supplementary Information 4). Three papers prior to 2009 discouraged the use of fluid restriction to achieve a decrease in body mass, whereas three papers after 2011 advised to consider the strategic use of 2–3% dehydration through fluid restriction or sweating for acute loss in body mass.

## Discussion

This scoping review aimed to summarise the dietary recommendations for altering body mass or composition in athletes, as well as evaluating changes over time, from expert consensus statements and position stands. A total of 73 papers across three types of publications were analysed, which were endorsed by 11 individual and three joint organisations, involving 328 experts from 25 countries. Despite the number of papers included, the range of sports and organisations represented was limited and many documents were not regularly updated. There were also inconsistencies in the evidence base, however, this comprehensive review synthesises four key recommendations that were consistently reported and should underpin evidence-based decision-making for a safer practice approach. They are: (1) involve qualified and experienced dietitians/nutritionists, (2) individualise outcome and dietary targets, (3) implement fundamental dietary practices—adapting calorie and protein intakes whilst avoiding unnecessary supplements and (4) ensure a minimum energy availability of 30 kcal/kg fat-free mass/day for health. Notably, almost half of papers omitted guidance on body mass and composition goal-setting and many lacked detail and specificity. Moving forward, given the importance of modifying behaviour to optimise performance and protect the health and wellbeing of athletes, recommendations for altering body mass or composition should clearly define target behaviours in specific and concrete terms. For example, what, who, when, where and how.

Nearly half of the papers in this review (*n* = 33) did not include recommendations on setting body mass or composition goals, optimal timelines for achieving these goals, or the most appropriate phases within the season for aligning these changes with training and competition objectives. For practitioners, a key initial step is to assess the appropriateness of targets, advise on suitable goals and challenge pre-conceived expectations or beliefs [3, 15]. Without evidence-informed guidance and qualified support, athletes and support personnel (e.g. coaches) may set unrealistic targets, compromising health and performance and increasing the risk of disordered eating and eating disorders [64] or the use of prohibited substances [14, 99]. Addressing the barriers to implementing evidence-informed guidelines is crucial, as elite sport practitioners from across the world report a lack of guidance in body mass and composition goal-setting [15], consistent with this review’s findings. Some papers provided guiding questions to ask athletes to evaluate target appropriateness [34, 53], such as: What is the maximum acceptable weight for you? What was the lowest weight you maintained without constant dieting? How did you derive your goal weight? At what weight and body composition do you perform best? However, more comprehensive, behaviourally specific recommendations are needed to ensure dietary advice is always preceded by appropriate body mass and composition target recommendations.

This review revealed that dietary recommendations for manipulating body mass and composition in athletes are not comprehensive enough. Once appropriate outcome targets are agreed upon with the athlete’s health team [15] and a nutrition assessment completed, nutritionists/dietitians are responsible for translating energy and nutrient requirements into tailored, evidence-based interventions that achieve the desired outcome [17]. Most papers focussed heavily on strategies to adjust calorie, protein and supplement intakes, but provided little guidance on other macro- and micronutrients. The language used was often vague, with phrases such as ‘adequate’ amounts, ‘enough’, ‘consume a surplus’ or ‘sufficient’ nutrients, which lack specificity and leave recommendations open to interpretation. This ambiguity relies heavily on practitioners, coaches and athletes to understand, interpret and implement advice correctly. Additionally, the narrow focus on calories, protein and supplements could lead athletes to overconsume protein at the risk of other nutrients, adopt restrictive or high risk dietary patterns and risk unintentionally violating anti-doping rules (e.g. through inadvertent exposure to prohibited substances) [79, 100, 101]. Developing more comprehensive, practice-oriented guidelines, such as those used in healthcare (e.g. the UK National Institute for Health and Care Excellence practice guidelines) by international sporting bodies could help to create clearer, actionable guidance for practitioners.

Despite this scoping review including 73 papers, many sports were not represented, and in general, recommendations were not developed by MDTs. Guidance was heavily skewed towards athletics, aquatics, team and weight-category sports. However, each sport and environment likely present different requirements for body mass and composition manipulation, given the variability of physiological demands across sports. In fact, a level of flexibility and depth is also necessary within recommendations, as not all guidance for one sport will be appropriate for all athletes from that sport. For example, guidance may need to be adapted for different positions in team sports, for different training demands of athletes from the same discipline or for athletes with specific dietary preferences such as vegetarianism. Researchers have started to implement this change with some nutrients in the last decade. For example, since 2014, protein recommendations have evolved from absolute portions for every athlete (e.g. 25g/meal) [59, 67], to relative portions (0.25–0.5 g/kg body mass/meal) [90, 91]. This highlights the need for more tailored and targeted guidelines alongside an inclusion of flexible recommendations that qualified practitioners can modify as appropriate for their individual athletes.

Generic recommendations for athletes were offered by 34 papers, meaning practitioners must translate these to their specific contexts, potentially leading to varied interpretations and sometimes inappropriate practice. For example, implementing a generic 500 kcal deficit to evoke 0.5 kg losses in fat mass per week is unlikely to cause negative health consequences for a 125 kg prop forward in rugby union, but might not be appropriate for a 60 kg high jumper with much lower energy availability. Developing nutrition guidelines with a MDT involving athletes could improve the effectiveness of interventions [102]. Yet this approach was not consistently evident in this review, and the professional backgrounds of experts was not often clear. Although rare, four papers (from the same conference) included a single athlete in their expert group to help develop the guidelines. A shift to this becoming the norm rather than the exception is encouraged and the field of sport and exercise nutrition could learn from the beneficial ‘patient and public involvement and engagement’ approaches that are commonplace in health settings [103]. Such engagement is now recommended or required by many major healthcare organisations when developing clinical practice guidelines (e.g. Guidelines International Network, Institute of Medicine and National Institute for Health and Care Excellence [104–106]). The field of sport and exercise nutrition is therefore encouraged to adopt a more intentional approach to drawing upon the lived experience of athletes (and practitioners) when designing and developing consensus recommendations to manipulate body mass and composition. With the target group in mind, some consensus conferences have also created athlete and coach friendly booklets such as the IOC Nutrition for Athletes [107] and FINA Nutrition for Aquatic Athletes [108]. Normalising the dissemination of accessible recommendations would be a positive development in the field, ensuring easier access to accurate information for organisations and athletes who do not have support from qualified nutrition professionals. Involving specialists from each discipline [15] and consulting with athletes in the production process may ensure guidelines are comprehensive, fit for purpose and encourage sustained adherence.

A total of 50 papers in this scoping review were not part of updated recommendations, making them standalone guidance documents that are likely to reflect outdated research over time. Through not limiting the search strategy by date, this review highlighted the progression of research and guidance since 1976. The change in dietary recommendations over decades, for example, advice on creatine supplementation (increase), daily protein targets (decrease) and fluid restriction (decrease), highlighted in this scoping review, shows the continued generation of new knowledge in the field, and the importance of regularly updating recommendations to reflect the latest research. Currently, the frequency of update to recommendations from international nutrition organisations is approximately 10 years (e.g. the three joint position statements from the American College of Sports Medicine, Dietitians of Canada and Academy of Nutrition and Dietetics on ‘Nutrition for athletic performance’ [4, 34, 53]). If this is standard, it could be argued that 32 papers in this review, published prior to 2014, require updating. In clinical practice, one out of five guidance documents are considered out of date after three years [109]. A periodical update is critical given yearly publication of sports nutrition papers has increased exponentially from approximately 200 in 2000 to 500 in 2010 and 3500 in 2021 [110]. Therefore, regular updates, more than once per decade, may be necessary moving forward. Consensus conferences were used to develop recommendations by 30 papers, which may present major challenges—logistically and financially—to regular updates. More participatory approaches that can be conducted online might help address the barriers of in-person consensus building events and also ensure a diverse range of stakeholders are involved to inform practice and policy-making [110].

Incorporating behavioural science into dietary recommendations could improve the development, description, implementation and evaluation of interventions aimed at manipulating athlete body mass and composition. Whilst it was beyond the scope of this review to systematically identify the ‘active components’ recommended alongside dietary targets in the form of behaviour change techniques (BCTs) [111–113], the review highlighted that such detailed analysis was impossible as target behaviours were unspecified in recommendations and guidelines. Previous research has noted that sport nutrition interventions primarily focus on education to increase nutrition knowledge and only achieve a 50% success rate in improving athlete dietary intake [114]. This limited success is potentially due to the restricted use of BCTs and the narrow application of behavioural theories within the field [113]. Given expert body mass or composition recommendations seek to modify athlete and practitioner behaviours, an absence of behavioural theory within such expert guidance may limit the successful implementation of these guidelines. Indeed, research points to a potential implementation gap given athletes are not meeting nutrition guidelines [115, 116]. Embedding behavioural science within the sport and exercise nutrition field could serve to advance the development of theory-informed and behaviourally targeted dietary interventions for effective athlete body mass and composition management.

### Strengths, Limitations and Future Directions

To our knowledge, this is the first scoping review of dietary recommendations for athletes by expert groups. The strengths of this study include the large number of recommendation papers reviewed, the inclusion of various international expert groups and the wide variety of athlete populations included. The comprehensive search strategy, spanning five databases without restrictions on date or sport, allowed for a thorough mapping of recommendation progression over time and across different sports.

However, this review is not without limitation. Although a systematic search of 73 consensus statements, position stands and practice guidelines was carried out, some recommendation papers may have been missed, specifically those that were challenging to identify as endorsed by expert organisations or developed from consensus conferences. To counteract this, a review of bibliographies was completed, identifying 87 additional papers. No quality appraisal was completed due to the purpose of scoping reviews being to map the existing literature regardless of methodological quality or risk of bias, thus the strength of individual recommendations was not assessed. Recommendations were extracted on the basis of author interpretation rather than explicit statements, necessitating a pilot extraction by two experienced and accredited sport nutritionists, L.D. and N.C. Following successful piloting, L.D. completed the full data extraction of all 73 papers. Recommendations for athletes with a disability were not included in this study as they are a population with a range of unique nutritional requirements that would be beyond the scope of this study.

Future research should produce practice guidelines specific to all sports, as this review highlighted that most recommendations were for general athlete populations, athletics, aquatics, team and weight category sports. The approach by international federations such as the International Association of Athletics Federation (now World Athletics), who host multiple expert nutrition consensus conferences [80, 117, 118], could be replicated, as recommendations are regularly revised to reflect the latest research. Guidelines should distinguish between male and female athletes and offer comprehensive advice on all macronutrients, micronutrients and dietary behaviours. Additionally, future recommendations should be underpinned by behavioural science, utilising theories of behaviour to inform advice, such as the Behaviour Change Wheel [102]. Scoping reviews of dietary recommendations to manipulate body mass or composition for athletes with a disability is also warranted. In addition, there is a body of evidence on manipulating the body mass or composition of military personnel that would also benefit from a scoping review.

## Conclusions and Implications

This scoping review provides a comprehensive summary of outcome targets and dietary recommendations for changing body mass or composition in male and female, non-disabled athlete populations. A total of 73 consensus statements, position stands and practice guidelines were identified, however, not all sports and disciplines were represented, and many were not regularly updated. Most recommendations lacked detailed guidance on target-setting, instead focussing on caloric intake, protein consumption and supplement use, with limited advice on other essential nutrients. Consistent advice included involving a qualified and experienced sport dietitian/nutritionist, and a MDT is essential to ensure that appropriate outcome targets, dietary behaviours and monitoring are implemented. Individual outcome targets with gradual rates of change and based on the sport, age, sex, genetics and an optimal power:mass ratio are essential. A calorie surplus or deficit should be implemented for increasing or decreasing body mass or composition, respectively, through individualised carbohydrate and fat modification. Intakes of protein should be on the higher end of the range with a high diet quality followed in both situations. Meals should be spread throughout the day, with appropriate carbohydrate and protein intakes before and after training sessions and assessment of the appropriateness and risks of supplementation considered. Adopting a participatory and sport-specific approach to regular guideline development, incorporating behavioural theories, including the broader MDT and consulting athletes, could result in athlete-centred recommendations that are more likely to achieve body mass and composition goals whilst protecting athlete health, wellbeing and performance.

## Supplementary Information

Below is the link to the electronic supplementary material.

Supplementary file1 (PDF 195 kb)

Supplementary file2 (PDF 72 kb)

Supplementary file3 (PDF 406 kb)

Supplementary file4 (PDF 544 kb)

## Declarations

### Availability of Data and Material

All extracted information is presented in the main text or supplementary material.
