# Supplementary material for: Dietary Recommendations for Body Mass and Composition Manipulation in Male and Female Athletes: a Scoping Review of Consensus Statements, Position Stands and Practice Guidelines from International Expert Groups
Source: Sports Med. 2025 Aug 21;55(10):2445–87. doi: 10.1007/s40279-025-02285-4 (PMC12513969; doi:10.1007/s40279-025-02285-4)
Supplement: Supplementary file 2 — Supplementary file2 (PDF 72 kb) [file 40279_2025_2285_MOESM2_ESM.pdf]

Dietary recommendations for body mass and composition manipulation in male and female athletes: A scoping review of consensus statements, position stands and practice guidelines from international expert groups

Lauren V. Delany[1,2], Nessan Costello[1], Ben Jones[1,3,4,5], Susan H. Backhouse[1]

[1] Carnegie School of Sport, Leeds Beckett University, Leeds, United Kingdom

[2] Sale Sharks Rugby Club, Manchester, United Kingdom

[3] England Performance Unit, Rugby Football League, Manchester, United Kingdom

[4] Division of Physiological Sciences, Department of Human Biology, Faculty of Health Sciences, The University of Cape Town and the Sports Science Institute of South Africa, Cape Town, South Africa

[5] Premiership Rugby Limited, London, United Kingdom

Corresponding author:

Lauren Delany

Carnegie School of Sport, Leeds Beckett University, Headingly Campus, Leeds, United Kingdom, LS6 3QU

l.delany@leedsbeckett.ac.uk

Supplementary Information 2: Electronic Search Strategy

SCOPUS Searched on 19/05/2023:

TITLE-ABS-KEY(athlet* OR player* OR sport*)

AND TITLE-ABS-KEY("body composition" OR anthro* OR weight OR mass OR fat OR muscle OR hypertrophy OR performance)

AND TITLE-ABS-KEY(nutrition OR diet OR food)

AND TITLE-ABS-KEY(guid* OR recommend* OR "position stand" OR consensus OR "position statement")

AND NOT TITLE-ABS-KEY(adoles* OR youth OR junior)

AND (LIMIT-TO (LANGUAGE , "English"))

AND (LIMIT-TO (DOCTYPE , "ar") OR LIMIT-TO (DOCTYPE , "re"))
