# Supplementary material for: Dietary Recommendations for Body Mass and Composition Manipulation in Male and Female Athletes: a Scoping Review of Consensus Statements, Position Stands and Practice Guidelines from International Expert Groups
Source: Sports Med. 2025 Aug 21;55(10):2445–87. doi: 10.1007/s40279-025-02285-4 (PMC12513969; doi:10.1007/s40279-025-02285-4)
Supplement: Supplementary file 3 — Supplementary file3 (PDF 406 kb) [file 40279_2025_2285_MOESM3_ESM.pdf]

Dietary recommendations for body mass and composition manipulation in male and female athletes: A scoping review of consensus statements, position stands and practice guidelines from international expert groups

Lauren V. Delany[1,2], Nessan Costello[1], Ben Jones[1,3,4,5], Susan H. Backhouse[1]

[1] Carnegie School of Sport, Leeds Beckett University, Leeds, United Kingdom

[2] Sale Sharks Rugby Club, Manchester, United Kingdom

[3] England Performance Unit, Rugby Football League, Manchester, United Kingdom

[4] Division of Physiological Sciences, Department of Human Biology, Faculty of Health Sciences, The University of Cape Town and the Sports Science Institute of South Africa, Cape Town, South Africa

[5] Premiership Rugby Limited, London, United Kingdom

Corresponding author:

Lauren Delany

Carnegie School of Sport, Leeds Beckett University, Headingly Campus, Leeds, United Kingdom, LS6 3QU

l.delany@leedsbeckett.ac.uk

Supplementary Information 3: Diet and supplement recommendations to increase athlete body mass or fat-free mass

STUDY	CALORIES			CARBOHYDRATE			PROTEIN			FAT			MICRONUTRIENTS			SUPPLEMENTS	FLUID
	TIME	TYPE	TOTAL	TIME	TYPE	TOTAL	TIME	TYPE	TOTAL	TIME	TYPE	TOTAL	TIME	TYPE	TOTAL		
Lemon (1991)	-	-	-	-	-	-	-	-	Additional dietary protein may be necessary.<br><br>Aim for a highly positive nitrogen balance.<br><br>Aim for protein intakes 1.2 - 1.7g/kg/day.<br><br>Higher intakes may be beneficial but would not be recommended.	-	-	-	-	-	-	-	-
Williams (1995)	-	-	-	-	-	-	-	-	-	-	-	-	-	-	-	Amino acid supplement not recommended.<br><br>Creatine not recommended.<br><br>HMB supplement not recommended.<br><br>Omega 3 supplement not recommended.<br><br>Vitamin B12 supplement not recommended.<br><br>Plant extract supplement not recommended.<br><br>Boron supplement not recommended.	-
Manore et al. (2000)	-	-	Consume sufficient energy.<br><br>Strength athletes: 44 to 50 kcal/kg body weight/d<br>Those in serious training > 50 kcal/kg body weight/d<br><br>Consume additional energy of 500 - 1,000 kcals per day	-	-	-	Consume protein after exercise.	-	Aim for adequate protein.<br><br>Additional protein is needed, and how much depends on:<br>- the type of exercise performed (endurance vs resistance)<br>- the intensity and duration of the activity<br>- possibly the sex of the participants.<br><br>Experienced male bodybuilders and strength athletes should aim for 1.6 - 1.7 g/kg BW/day<br><br>No need to consume more than recommendations.<br><br>No data for female strength athletes.	-	-	-	-	-	-	-	-

STUDY	CALORIES			CARBOHYDRATE			PROTEIN			FAT			MICRONUTRIENTS			SUPPLEMENTS	FLUID
	TIME	TYPE	TOTAL	TIME	TYPE	TOTAL	TIME	TYPE	TOTAL	TIME	TYPE	TOTAL	TIME	TYPE	TOTAL		
Loucks (2004)	-	-	Manage carbohydrate, protein and fat balance to achieve a sport-specific body size and body composition.	Consume plenty of carbohydrates after exercise to elevate insulin concentrations to promote the uptake of amino acids and the synthesis of protein by muscle.	-	Consume sufficient carbohydrate to fuel anabolic processes.	-	Consume complete protein.<br><br>Add sufficient carbohydrate to protein consumption.	Induce a positive protein balance by consuming adequate amounts of complete protein.	-	-	-	-	-	-	-	-
Maughan et al. (2004)	-	-	-	-	-	-	-	-	Much higher amounts of protein are not needed.	-	-	-	-	-	-	Glutamine, branched-chain amino acids, leucine, lysine, arginine, and ornithine supplements not recommended.<br><br>Creatine is recommended.<br>Creatine loading associated 1-2 kg body mass gain within 4-5 days but may be more.<br>Initial loading dose: 20g/day for 4-5 days<br>Followed by maintenance dose of 1-2g/day<br>Alternative approach: 10g/day for 3-4 days if co-ingestion with sufficient carbohydrate.<br><br>HMB supplement not recommended.<br><br>Chromium supplement not recommended.<br><br>Vanadium supplement not recommended.<br><br>Boron supplement not recommended.<br><br>Herbal extracts not recommended.	-
Spriet et al. (2004)	-	-	-	-	-	-	-	-	-	-	-	-	-	-	-	Creatine not recommended.	-
Tipton et al. (2004)	-	-	Consume a hyperenergetic diet	-	-	-	Take advantage of timing of protein intake.<br><br>Varying the timing of protein and amino acid intake.<br><br>Consuming amino acids after resistance exercise is critical.	Vary the type of protein and amino acid consumed.	Aim to increase protein or amino acid intake.<br><br>Tailor protein intake strategy to:<br>- the desired change<br>- functional needs of the sport<br>- positional requirements<br>- athlete individual needs.<br><br>Recommendation unclear: Consume relatively high protein intake: 2g/kg BW/day.<br><br>No need to consume more than 2g/kg body weight/day.	-	-	-	-	-	-	-	-

STUDY	CALORIES			CARBOHYDRATE			PROTEIN			FAT			MICRONUTRIENTS			SUPPLEMENTS	FLUID
	TIME	TYPE	TOTAL	TIME	TYPE	TOTAL	TIME	TYPE	TOTAL	TIME	TYPE	TOTAL	TIME	TYPE	TOTAL		
Burke et al. (2006)	Consuming small, frequent meals and snacks throughout the day to assist to meet high energy needs.	Avoid excessive intake of low energy-dense foods when these foods reduce appetite or impair total food intake, as this may impact ability to meet high energy needs.<br><br>Making use of energy- and nutrient-dense fluids such as fortified milk drinks and liquid meal supplements as this may assist to meet high energy needs.	Consider, adjust and individualise energy intakes when manipulating muscle mass and body fat.<br><br>Consume adequate energy. However, Information on optimal intake of energy is lacking.<br><br>Maybe increase in energy intake is needed.	-	Avoid excessive intake of fibre-rich foods when these foods reduce appetite or impair total food intake, as this may impact ability to meet high energy needs.	-	Consuming carbohydrate and protein before, during and after resistance training may enhance adaptations.	-	-	-	-	-	-	-	-	-	-
Hespel et al. (2006)	-	-	-	-	-	-	-	-	-	-	-	-	-	-	-	Protein hydrolysate/protein supplement recommended. Amino acid supplement recommended.<br><br>Creatine recommended. Creatine loading associated 1-3 kg body mass gain within 3 - 4 days.<br>Initial loading phase: 15-20g/day for 4-7days<br>Followed by maintenance dose of 2-5g/day<br>Every 8-10 weeks of supplementation include wash-out period of at least 4 weeks.<br>Avoid co-ingestion with caffeine.<br>Co-ingestion with carbohydrate/amino acid mixture.<br>Consume in early recovery (1 - 3 h) after strength and power exercise sessions.<br><br>HMB supplement maybe recommended.	-
Buford et al. (2007)	-	-	-	-	-	-	-	-	-	-	-	-	-	-	-	Creatine recommended.<br>Creatine monohydrate loading associated 1-2 kg body mass gain in first week.<br>In long-term studies, subjects taking CM typically gain about twice as much body mass and/or fat free mass (i.e., an extra 2 to 4 pounds of muscle mass during 4 to 12 weeks of training) than subjects taking a placebo.<br>Quickest method: Initial loading phase: 0.3 g/kg/day for at least 3 days<br>Followed by maintenance phase of 3-5g/day<br>Alternative approach: 2-3g/day for 3-4 weeks<br>Alternative approach: 6g/day for 12 weeks<br><br>Beta alanine if combined with creatine monohydrate – maybe recommended	-
Campbell et al. (2007)	-	-	-	-	-	-	Aim for appropriately timed		Aim for a protein intake 1.4 -							Whey protein supplement if not able to access from	-

STUDY	CALORIES			CARBOHYDRATE			PROTEIN			FAT			MICRONUTRIENTS			SUPPLEMENTS	FLUID
	TIME	TYPE	TOTAL	TIME	TYPE	TOTAL	TIME	TYPE	TOTAL	TIME	TYPE	TOTAL	TIME	TYPE	TOTAL		
							protein intakes.<br><br>A strategically planned protein intake regimen timed around physical activity is integral. To increase muscle protein synthesis, consume protein and carbohydrate before and/or after exercise.<br><br>Ensure a high level of blood amino acids after resistance exercise.		2.0g/kg/day. Athletes completing endurance exercise at the lower end, intermittent exercise in the middle and strength/power exercise at the upper end of this range							whole foods is recommended.<br><br>Branched chain amino acid supplement, only if not able to access from whole foods or whey protein supplement is recommended	
Houtkooper et al. (2007)	-	-	Consume adequate/enough energy	Time carbohydrate consumption to provide adequate fuel for energy demands and to spare protein for muscle growth.		Consume adequate carbohydrate as fuel to increase muscle mass through strength training.	Time adequate protein intake to meet needs.<br><br>Aim for adequate protein intake on rest days.<br><br>Consume protein close to the start and/or end of exercise.	-	Aim for adequate protein intake on rest days.<br><br>Individualise recommendations for protein.<br><br>Ensure protein consumption does not interfere with consumption of other nutrients especially carbohydrate.	-	-	-	-	-	-	-	-
Manore et al. (2007)	-	-	-	-	-	-	-	-	-	-	-	-	-	-	Assess the diets of active females for adequate zinc intake, which plays a role in growth of muscle tissue.	-	-
Maughan et al. (2007)	-	-	-	-	-	-	-	-	-	-	-	-	-	-	-	Glutamine, branched-chain amino acids, leucine, lysine, arginine, and ornithine supplement not recommended.<br><br>Creatine maybe recommended. Creatine loading associated 1-4 kg body mass gain.<br>Initial loading phase: about 20g/day in 4 divided doses for 4-6 days<br>Followed by maintenance dose of 2-5g/day<br>Interrupt 4-6 weeks of supplementation with a break of about 4 weeks.<br><br>Caffeine and buffering agents maybe recommended.<br><br>Any athletes looking to use supplements for increasing weight, should gain assurances regarding quality control of supplement manufacture to ensure freedom from contamination with toxic or prohibited components.	-

STUDY	CALORIES			CARBOHYDRATE			PROTEIN			FAT			MICRONUTRIENTS			SUPPLEMENTS	FLUID
	TIME	TYPE	TOTAL	TIME	TYPE	TOTAL	TIME	TYPE	TOTAL	TIME	TYPE	TOTAL	TIME	TYPE	TOTAL		
Stellingwerff et al. (2007)	-	-	-	Consume carbohydrate both during and after resistance exercise.	-	Consume 0.5g carbohydrate/ kg/ hr during resistance exercise, for muscle hypertrophy.<br><br>Consume 0.5g carbohydrate/ kg/ hr after resistance exercise, for muscle hypertrophy.	Consume dietary essential amino acids during and 2 hours after exercise.<br><br>To start protein synthesis, consume 1-4g carbohydrate/kg body weight and 0.1g essential amino acids/kg body weight within 4 hrs after exercise.	-	Essential amino acids 0.1g/kg body weight	-	-	-	-	-	-	-	-
Tipton et al. (2007)	-	-	Energy intake is important/crucial/should be carefully considered.<br><br>Aim for energy balance.<br><br>Increase energy intake.	-	-	Consume sufficient carbohydrate to maintain glycogen during training.	Timing of protein must be considered in designing optimal nutrition strategies.<br><br>To increase muscle mass, consume protein or a source of amino acids and carbohydrates after exercise.	Consider the type of protein.<br><br>Consume a source of amino acids from intact protein or free amino acid mixture.<br><br>Co-ingestion of protein with other nutrients in a meal is an influencing factor.	Protein intakes are important/ should be considered.<br><br>A wide range of protein intakes is appropriate.<br><br>Estimated needs: 1.2 - 1.5g/kg/day.<br><br>Recommending increased protein intake not needed if well-chosen diet. If increasing energy intake then portion of this could be protein.<br><br>No need to consume more than 2g/kg/day, but those with years of training may have different needs.<br><br>Optimal amounts of amino acids yet to be determined.<br><br>Positive protein balance can be achieved with 12g protein.	-	-	-	-	-	-	Protein supplement not recommended as no better than food.<br>Amino acid supplement not recommended as no better than food.<br><br>Creatine recommended but individual responses vary.<br>Creatine loading associated with increased body mass.<br>Sprinters must consider the extra weight gain associated with creatine use.<br>Positive effect may diminish after 8-10 weeks.<br>Initial loading phase: 15-20g/day for 4-7days<br>Followed by maintenance dose of 2-5g/day<br>Every 8-10 weeks of supplementation include wash-out period.	-
Kerksick et al. (2008)	-	-	-	Consume carbohydrates alone, or with protein during resistance training, to increase muscle cross-sectional area.	-	-	Consuming amino acids after resistance exercise at a range of different time points is beneficial.<br>Consume amino acids, primarily essential amino acids immediately after exercise through 3 hours after.<br><br>To improve body composition/ stimulate protein synthesis, consume 30-40g high-glycaemic carbohydrate and 6-20g essential amino acids both before and within 3 hrs after exercise or resistance training.	Regularly consume various protein sources with carbohydrate and this may favourably impact body composition.<br><br>Adding carbohydrate to essential amino acid intake may enhance effect.<br><br>Choose whey protein instead of casein protein with carbohydrate after exercise to increase protein synthesis.	Increase the concentration and availability of amino acids in the blood is an important consideration.<br><br>20-75g protein<br>6 - 40g essential amino acids	-	-	-	-	-	-	Protein and carbohydrate supplement recommended.<br>Amino acid or protein supplement, alone or in combination with carbohydrate recommended.<br><br>Creatine recommended.<br>Add creatine to a post-exercise regimen of carbohydrate and protein.	-

STUDY	CALORIES			CARBOHYDRATE			PROTEIN			FAT			MICRONUTRIENTS			SUPPLEMENTS	FLUID
	TIME	TYPE	TOTAL	TIME	TYPE	TOTAL	TIME	TYPE	TOTAL	TIME	TYPE	TOTAL	TIME	TYPE	TOTAL		
							To increase lean mass and improve body fat %, consume 50-75g carbohydrates and 20-75g protein during resistance training.										
New Zealand Dietetics Association (2008)	-	-	Increase energy intake to meet training needs, especially with carbohydrates.	-	-	Increase energy and carbohydrate intake to prevent protein being oxidised, to increase muscle mass.	Focus on the timing of protein as this is most important.	-	Adequate protein intake is necessary.<br><br>No benefit of higher-protein intakes.<br><br>Maximum protein intake is 2g/kg body mass/day and no benefit to higher intakes.	-	-	-	-	-	-	-	-
Rodriguez et al. (2009)	-	-	Consume sufficient energy.	-	-	-	Consume protein after exercise.<br><br>Consume protein or amino acids near strength and endurance exercise.	Choose intact, high-quality protein e.g. whey, casein or soy.	Protein intakes in excess of RDA during resistance exercise may be needed.<br><br>Strength-trained athletes should aim for approx. 1.2 - 1.7g/kg/day	-	-	-	-	-	Athletes may require greater intakes of micronutrients, to build lean body mass.<br><br>Ensure adequate intakes of B vitamins, to build muscle tissue.<br><br>Consider zinc intake as it plays a role in growth and building muscle tissue.	Protein supplement is no better than food so not recommended<br>Amino acid supplement is no better than food so not recommended	-
Holway et al. (2011)	-	-	-	-	-	-	To accelerate protein synthesis, consume 1.2g carbohydrate/ kg/ day and protein after a match, from either food or supplements.	Consume carbohydrate and protein together after matches.	-	-	-	-	-	-	-	-	-
La Bounty et al. (2011)	-	-	Consider energy intake and expenditure when optimising body composition.<br><br>Aim for hyperenergetic eating patterns.	-	-	-	-	-	20-30g protein/meal<br>10 - 15g essential amino acids/peal	-	-	-	-	-	-	-	-
Meyer et al. (2011)	-	-	-	-	-	-	To manage lean body mass, consume 1.2-1.5g carbohydrate/kg/hr, starting within the first 30 mins and with 15-20g protein, after intense training.	Consume carbohydrate and protein together after intense training.	-	-	-	-	-	-	-	-	-
Phillips et al. (2011)	-	-	-	-	-	-	Spread protein intake across the day in 3-4 equal meals. There are benefits of repeated periods of positive protein balance<br><br>A 'window of anabolic opportunity' is not well defined,	Add carbohydrate to protein to optimise effect.<br><br>Consume leucine e.g. milk proteins and possibly other branched-chain amino acids.<br><br>Consume milk	Protein intake can enhance rates.<br><br>Aim for protein intakes of 1.3 - 1.8g/kg/day.<br><br>Training status may impact recommendations as experienced athlete require less.	-	-	-	-	-	-	High-leucine whey recommended vs soy protein supplement.<br>Isolated leucine not recommended vs high quality protein.	-

STUDY	CALORIES			CARBOHYDRATE			PROTEIN			FAT			MICRONUTRIENTS			SUPPLEMENTS	FLUID
	TIME	TYPE	TOTAL	TIME	TYPE	TOTAL	TIME	TYPE	TOTAL	TIME	TYPE	TOTAL	TIME	TYPE	TOTAL		
							therefore consume protein and/or amino acids as soon as possible after exercise.<br><br>No benefit of consuming protein before exercise or during aerobic exercise.	proteins rather than isolated soy protein after resistance exercise.<br><br>Consume higher leucine content and rapidly digesting proteins.	Consume more protein during periods of high frequency/ intensity training.<br><br>Optimum intake 20-25g protein. This may be lower for lighter athletes.								
Slater et al. (2011)	-	-	Consider total energy intake.	-	-	-	Consider daily distribution of protein intake, in particular in relation to training.<br><br>Consume high biological protein at no more than 5-6 times daily.<br><br>Consume protein after exercise.<br><br>Consume high biological value protein after resistance exercise.	Consider the source of protein.<br><br>Consume high biological value protein after resistance exercise.	Optimize protein intakes.<br><br>20g protein<br>8-10g essential amino acids	-	-	-	-	-	-	-	-
Buell et al. (2013)	To gain lean-muscle mass, eat frequently and ensure adequate calories.	-	Consume adequate calories.	-	-	Aim for a carbohydrate intake of at least 6g/ kg/ day, to gain lean muscle mass.	-	-	Maximum protein intake of 1.7g/kg/day	-	-	-	-	-	-	-	Ensure sufficient hydration during activity to allow muscles to work hard.
Sundgot-Borgen et al. (2013)	-	-	Consume a modest positive energy balance.	-	-	-	-	-	-	-	-	-	-	-	-	-	-
Team Physicians et al. (2013)	Optimize timing and distribution of energy intake and avoid skipping meals.	More calories and carbohydrates through food selection; provides examples of foods and drinks to switch to, to increase calorie intake.<br><br>Increase energy intake; provides examples of high energy options.<br><br>Carbohydrate/calorie food to increase energy intake. Also provide examples of low volume and convenient foods to increase energy intake.	Adjust energy intake to consume the recommended energy intake.<br><br>Aim for a positive energy balance through adjusting energy intake.	-	-	-	Consume protein after exercise.	-	Follow recommended protein intakes through the diet.<br><br>Aim for protein intakes at upper end of the range: 0.55 - 0.77g/lb	-	-	-	-	-	-	Protein supplement not recommended.<br><br>Amino acid supplement not recommended.	Maintain proper hydration.
Wilson et al. (2013)	-	-	-	-	-	-	-	-	-	-	-	-	-	-	-	Leucine supplement with resistance training recommended.<br><br>HMB recommended.<br>1) in untrained individuals, HMB can enhance muscle hypertrophy and dynamic strength in as little as three weeks;<br>2) for trained individuals it is important to realize that adaptations occur at a slower rate than in untrained	-

STUDY	CALORIES			CARBOHYDRATE			PROTEIN			FAT			MICRONUTRIENTS			SUPPLEMENTS	FLUID
	TIME	TYPE	TOTAL	TIME	TYPE	TOTAL	TIME	TYPE	TOTAL	TIME	TYPE	TOTAL	TIME	TYPE	TOTAL		
																individuals. For this reason, HMB will likely be most beneficial over longer training durations (> 6 weeks) in trained individuals. Presently, available literature suggests 38 mg/kg body mass daily, divided into two to three servings provides an adequate amount of HMB to enhance adaptive processes in muscle.	
Benardot et al. (2014)	-	-	Maintain energy balance	-	-	-	To enhance muscle protein synthesis, consume a carbohydrate- and protein-rich snack immediately after exercise and ensure adequate protein.<br><br>Adapt recovery recommendations to the needs of individual divers.	Choose high quality protein.	20-30g protein/meal.	-	-	-	-	-	-	-	-
Cox et al. (2014)	-	-	-	-	-	Daily carbohydrate requirements between 4-8 g/kg body mass/day.<br><br>A higher carbohydrate intake may be needed to maintain a positive energy balance, to gain lean muscle mass.	Consume protein immediately after a workout.	Choose a fast-acting protein after workouts e.g. whey.	20-30g protein	-	-	-	-	-	-	-	-
Derave et al. (2014)	-	-	-	-	-	-	Ingestion of protein (about 20-25g) around exercise.	-	20-25g protein	-	-	-	-	-	-	-	-
Mujika et al. (2014)	-	-	Periodize calories and macronutrient intake towards desired body composition change.<br><br>Use an individualized approach to fuel needs based on each athlete's body size and composition and each athlete's specific training and competition program.	If needed, consume carbohydrate during resistance training sessions.	-	Aim to complete resistance training with enough carbohydrate stores and consume carbohydrate during session, to build muscle mass.	Spread the intake of high-quality protein over the day through meals/snacks every 3-4 hours. Key time points include after important training session and before sleep.<br><br>Protein intake in conjunction with exercise. Consume protein after resistance and endurance training, or after important training sessions.<br><br>To support muscle anabolism, the optimal choice is to ingest protein soon after exercise or alternatively resume normal post-exercise feeding pattern.	Choose a high-quality protein with sufficient essential amino acids/leucine.	Ingestion of a source of amino acids to raise blood essential amino acid levels.<br><br>0.25-0.30 g protein/kg body mass	-	-	-	-	-	-	-	-
Robertson et al. (2014)	-	-	-	-	-	Recommended carbohydrate intakes 5 - 7g /kg /day. Adjust carbohydrate targets depending	Timing of protein is critical.<br><br>To influence muscle protein synthesis, ensure	To influence muscle protein synthesis, ensure consumption of carbohydrates	-	-	-	-	-	-	-	-	-

STUDY	CALORIES			CARBOHYDRATE			PROTEIN			FAT			MICRONUTRIENTS			SUPPLEMENTS	FLUID
	TIME	TYPE	TOTAL	TIME	TYPE	TOTAL	TIME	TYPE	TOTAL	TIME	TYPE	TOTAL	TIME	TYPE	TOTAL		
						on body composition goals.	consumption of carbohydrates and protein both before and after exercise.	and protein both before and after exercise.									
Shaw et al. (2014)	-	-	Adapt total energy intake according to physique manipulation goals.	-	-	Adapt carbohydrate intake according to physique manipulation goals.	Spread the intake of protein over the day in similar amounts, at 4-5 opportunities through meals and snacks and before bed.<br><br>Consume high biological value protein soon after pool sessions, key workouts, resistance exercise sessions or a race.	Choose high biological value protein after pool sessions, resistance exercise or a race.	0.3 g protein/kg fat free mass - Unclear<br>0.3g/kg body mass approx. 20-25g protein	-	-	-	-	-	-	-	-
Thomas et al. (2016)	-	-	Aim for an adequate and appropriate energy intake as this assists in manipulating body composition. Over 4-12 weeks.<br>Do not misuse alcohol as this can compromise the management of body composition.	-	-	Carbohydrate recommendations are 3-10 g/kg BW/day and up to 12 g/kg BW/d for extreme and prolonged activities. Adapt based on body composition goals.	Aim for a well-timed protein intake.<br><br>Consume protein during or after exercise.<br><br>Consume protein immediately after exercise and in the 24-hour period after exercise.	Choose high quality dietary protein.<br><br>Choose milk-based protein after resistance exercise.<br><br>Protein options include whole milk, lean meat, and dietary supplements, some providing the isolated proteins whey, casein, soy, and egg.	20-30g total protein<br>Approx. 10g essential amino acids	-	-	Individualize fat intake based on body composition goals.	-	-	-	Creatine maybe recommended.	-
Aragon et al. (2017)	No advantage of intermittent caloric restriction over daily caloric restriction to improving body composition.	-	Aim for a sustained caloric surplus.<br><br>Untrained subjects and those with high level of NEAT - aim for a larger caloric surplus.<br><br>Advanced trainees should aim for a smaller caloric surplus.<br><br>Variation in individual responses for both untrained and advanced trainees	-	-	-	-	-	Consider increasing dietary protein beyond current recommendations for athletic populations may improve body composition.<br>Protein requirements in athletic populations is 1.4-2.0g/kg/day.	-	-	-	-	-	-	-	-
Jager et al. (2017)	-	Increase calories through small snacks between meals of both complete protein and carbohydrates	Increase calories.	-	-	-	The frequency and pattern of optimal protein doses is a key determinant.<br><br>Timing of protein intake in between the regular 3 meals a day may be additive.<br><br>Consume protein before or after resistance exercise. To maximise rates of muscle protein synthesis, consume carbohydrate and amino acids before exercise.<br>Consume protein or various forms of amino acids in the hour immediately	Consume rapidly digested, high quality proteins that contain high proportions of essential amino acids and adequate leucine.<br><br>Consume whole food sources of protein that contain all of the essential amino acids.<br><br>High-quality animal and dairy-based proteins e.g. meat, milk, eggs, and	Increasing dietary protein can promote favourable adaptations.<br><br>Aim for protein intakes of 1.4 - 2.0g/kg body weight/day.<br>Higher intake of protein (>3g/kg/day) may benefit body composition.<br><br>0.25g/kg body weight/serving or 20 - 40g protein. Higher end of range may be needed in the elderly.	-	-	-	-	-	-	Intact protein, whey protein recommended vs casein.<br><br>Leucine supplement alone not recommended.<br><br>Essential amino acids and leucine supplement recommended.	-

STUDY	CALORIES			CARBOHYDRATE			PROTEIN			FAT			MICRONUTRIENTS			SUPPLEMENTS	FLUID
	TIME	TYPE	TOTAL	TIME	TYPE	TOTAL	TIME	TYPE	TOTAL	TIME	TYPE	TOTAL	TIME	TYPE	TOTAL		
							before or during exercise.<br>Aim for a protein distribution pattern of 20-25g feed every 3 hours after resistance exercise.<br>Consume carbohydrates and protein or essential amino acids during endurance and resistance exercise.<br><br>Consume 30-40g casein protein before 30 mins sleep or 2 hours after last meal.	cheese are better options than vegetarian/plant proteins after resistance exercise.<br><br>Meat-based diets may be beneficial.<br><br>Consider consuming a combination of higher quality protein sources (dairy, egg, and meat sources).<br><br>Protein supplement options include free form essential amino acids, soy, milk, whey, caseinate, and other protein hydrolysates.<br><br>Elderly individuals should choose intact protein feedings of appropriate amounts, as opposed to free amino acids.	10-12g essential amino acids or 6 - 15g - unclear<br>1 - 3g leucine								
Kerksick et al. (2017)	Consider the timing of energy intake and ratio of macronutrients.	-	-	-	-	-	Consuming 20-40g high-quality protein every 3-4 hours across the day is favourable.<br><br>Consider the size and timing of pre-exercise meal as this may impact the need for post-exercise protein intake.<br><br>Consume high-quality protein after exercise, immediately to 2 hours after.<br><br>To increase muscle protein synthesis, consume carbohydrate and protein either before or after exercise.<br><br>Consume approx. 30-40g casein protein 30 mins before sleep or 2 hours after last meal.	Consume essential amino acids in free form or as part of a protein bolus.<br><br>Consume high quality protein throughout the day and after exercise.<br><br>Consume approx. 30-40g casein protein 30 mins before sleep or 2 hours after last meal.	Protein metabolism across the day is impacted by:<br>- exercise type<br>- training status and<br>- consumption of mixed macronutrient meals.<br><br>10 - 12g essential amino acids<br>20 - 40g protein bolus<br>0.25-0.40 g/kg/serving<br>Casein protein approx. 30 - 40g before bed	-	-	-	-	-	-	Whey protein supplement recommended.<br><br>Essential amino acids in free form or in protein bolus recommended	-
Kreider et al. (2017)	-	-	-	-	-	-	-	-	-	-	-	-	-	-	-	Creatine monohydrate recommended.	-

STUDY	CALORIES			CARBOHYDRATE			PROTEIN			FAT			MICRONUTRIENTS			SUPPLEMENTS	FLUID
	TIME	TYPE	TOTAL	TIME	TYPE	TOTAL	TIME	TYPE	TOTAL	TIME	TYPE	TOTAL	TIME	TYPE	TOTAL		
																The types of sport events in which creatine supplementation has been reported to benefit for increased Body Mass/Muscle Mass: American Football, Bodybuilding, Combat Sports (e.g., MMA, Wrestling, Boxing, etc.), Powerlifting, Rugby, Track/Field events (Shot put; javelin; discus; hammer throw), Olympic Weightlifting.<br>Quickest method: Initial loading phase: 5g/day (or approx. 0.3 g/kg body weight), 4 times daily for 5-7 days<br>Followed by maintenance phase of 3-5g/day<br>Larger athletes may need 5-10g/day in maintenance phase<br>Alternative approach: 3-5g/day for 3-4 weeks<br>Co-ingestion with carbohydrate or carbohydrate and protein.	
Maughan 2018	-	-	-	-	-	-	Spread protein intake across the day in 3-4 meals, and close to exercise.<br><br>Consume protein containing meal close to exercise, in particular after exercise.	-	Optimal daily dose of 1.6 - 2.2g/kg/day.<br><br>0.3 - 0.5g/kg/meal protein.	-	-	-	-	-	-	Protein supplement powder, ready-to-drink liquid; protein-rich bar recommended.<br>Provides 20-50 g protein in a single serve from high-quality types of animal (whey, casein, milk, egg) or vegetable (e.g., soy) protein<br>Isolated protein supplement from various sources (e.g. whey or soy) recommended.<br>Leucine supplement, no long-term trials showing efficacy – not recommended.<br><br>Creatine recommended.<br>Creatine loading associated 1-2 kg body mass gain.<br>May be detrimental for endurance performance or in events where body mass must be moved against gravity (e.g., high jump, pole vault) or where athletes must achieve a specific body mass target.<br>Initial loading phase: 20g/day in 4 equal doses for 5-7 days<br>Followed by maintenance phase of 3-5g/day in a single dose<br>Co-ingestion with protein/carbohydrate mix (approx. 50 g of protein)<br>No negative health effects are noted with long-term use (up to 4 years) when appropriate loading protocols are followed.<br><br>HMB supplement not recommended.<br>Omega3 supplement at 2g/day recommended but may not benefit when protein is ingested after exercise in recommended amounts.	-

STUDY	CALORIES			CARBOHYDRATE			PROTEIN			FAT			MICRONUTRIENTS			SUPPLEMENTS	FLUID
	TIME	TYPE	TOTAL	TIME	TYPE	TOTAL	TIME	TYPE	TOTAL	TIME	TYPE	TOTAL	TIME	TYPE	TOTAL		
Burke et al. (2019a)	-	-	Focus on adapting energy balance (dietary energy intake and total daily energy expenditure) to support changes in body composition.	-	-	-	Periodize protein intake for physique manipulation.	-	Do not set protein targets to achieve the absence of insufficiency as this is not appropriate for competitive athletes who need to achieve a certain physique.<br><br>Optimum intake of 1.3 - 1.7g/kg body mass/day.<br><br>No need to consume more than 2.5g/kg body mass/day of protein.<br><br>0.3 - 0.4g/kg body mass/meal protein	-	-	-	-	-	-	-	-
Close et al. (2019)	-	-	-	-	-	-	-	-	-	-	-	-	-	-	-	Creatine monohydrate recommended.<br>Suggested dose: 20 g/day for 5 days followed by 5 g/day thereafter.	-
Desbrow et al. (2019)	-	-	-	-	-	-	Spread protein intake across the day in 3-4 feeds.<br><br>Consume a meal containing high-quality protein after training.	Choose whole food sources of protein.<br><br>Consume a meal containing high-quality protein after training.	Aim for a protein target a 1.5 - 1.6g/kg/day.<br><br>Approx. 0.4g/kg protein/portion.	-	-	-	-	-	-	-	-
Jager et al. (2019)	-	-	-	-	-	-	-	-	-	-	-	-	-	-	-	Probiotic supplementation is not recommended for altering body composition in athletes.	-
Lis et al. (2019)	-	-	Insufficient evidence to support fasting compared with conventional techniques for improving body composition.	-	-	-	-	-	-	-	-	-	-	-	-	-	-
Peeling et al. (2019)	-	-	-	-	-	-	-	-	-	-	-	-	-	-	-	Creatine monohydrate recommended.<br>Creatine loading associated 1-2 kg body mass gain. May be counterproductive for weight-sensitive events, such as jumps and distance races.<br>A low-dose approach that avoids the CM 'loading phase' may avoid such issues.<br>Initial loading phase: 20g/day in 4 equal doses for 5-7 days<br>Followed by maintenance phase of 3-5g/day in single dose<br>Alternative approach: lower dose 2-5 g/day, for approx. 4 weeks.<br>Co-ingestion with a carbohydrate/protein mixture (approx. 50 g of protein)	-
Slater et al. (2019)	-	-	Consider total energy intake.	-	-	-	Consider daily distribution of protein intake.<br>Consume protein containing meals every 3-5 hours.	Consume high biological value protein.	General guidelines to consume 1.6 - 2.2g/kg/day.<br><br>0.4g/kg/meal protein.	-	-	-	-	-	-	Creatine recommended.<br>All sprint events may benefit from creatine supplementation but potentially greatest benefit for shorter sprints (100 m, 200 m).<br>During competition - sprinters must weigh potential performance benefits versus energetic costs associated with extra	-

STUDY	CALORIES			CARBOHYDRATE			PROTEIN			FAT			MICRONUTRIENTS			SUPPLEMENTS	FLUID
	TIME	TYPE	TOTAL	TIME	TYPE	TOTAL	TIME	TYPE	TOTAL	TIME	TYPE	TOTAL	TIME	TYPE	TOTAL		
																body mass and fluid retention that may occur with creatine monohydrate supplementation.	
Stellingwerff et al. (2019)	-	-	Macro periodisation of energy availability is needed to optimise body composition periodisation.<br><br>Aim for appropriate manipulation of energy availability.	-	-	-	Protein is important when planning within-day macronutrient periodization.<br><br>Protein is important to optimise acute recovery.	-	Optimize dietary and supplementation of protein.	-	-	-	-	-	-	Protein supplement maybe recommended.<br><br>Creatine maybe recommended.<br><br>Beta alanine maybe recommended	-
Tiller et al. (2019)	-	-	Careful consideration of weekly energy requirements of both training and recovery is recommended to achieve an individual goal of weight loss or gain.	-	-	-	Optimal strategy includes consuming approx. 20g protein every 3 waking hours, which is better than pulse-feeing of 10g every 1.5 hours or bolus-feeding of 40g every 6 hours.<br><br>Consume protein before sleep.	-	Aim for protein intakes of approx. 1.6 - 2.1 g/kg/day.<br><br>No need to consume more than 2.1g/kg/day<br><br>20g protein portion.	-	-	-	-	-	-	Whey protein supplement recommended.<br><br>Leucine with whey supplement recommended.	-
Witard et al. (2019)	-	-	Aim for a positive energy balance.<br><br>Aim for an energy surplus of approx. 500 kcal per day.<br><br>Athletes with a long history of heavy strength training should carefully consider and monitor the excess energy intake.	-	-	-	Protein timing in relation to training, sleep, and other nutrients is an influencing factor.<br><br>Protein meal pattern is an influencing factor. Distribute daily protein intake evenly across the day in 4-5 equally spaced servings.<br><br>Consume a slow-releasing, protein-rich, snack before bed.	Type of protein is an influencing factor.<br><br>Might not need an emphasis on protein-containing foods.<br><br>Co-ingestion of protein with other nutrients is an influencing factor.	Aim for dietary protein intakes above daily requirements of 0.8-1g/kg body mass/day as this benefits manipulating body composition.<br><br>Broad guideline to consume 1.3 - 1.6 or 1.7 g/kg body mass/ day - unclear which.<br>No need to consume more than 1.6g/kg body mass/day.<br><br>Excessive protein consumption could interfere with consumption of other nutrients especially carbohydrate.<br><br>0.3 - 0.4g/kg body mass/meal protein.<br>Optimal 'real world' serving 0.4 - 0.5g/kg BM/meal.	-	-	-	-	-	-	Protein supplement only for convenience is not recommended.<br>High-leucine whey vs lower-leucine soy or micellar casein or wheat recommended.<br><br>Amino acid supplement, only for convenience not recommended.<br><br>Branched chain amino acids not recommended	-
Konig et al. (2020)	-	-	-	-	-	-	-	Adequate quality of protein intake can have a positive effect on improving body composition<br><br>Consume high quality proteins and amino acids.<br><br>No protein	Adequate quantity of protein and amino acid intake can have a positive effect on improving body composition.<br><br>Increasing protein intake in the diet may be helpful for sports where periodic phases focus on increasing muscle mass or reducing	-	-	-	-	-	-	Protein supplement not recommended	-

STUDY	CALORIES			CARBOHYDRATE			PROTEIN			FAT			MICRONUTRIENTS			SUPPLEMENTS	FLUID
	TIME	TYPE	TOTAL	TIME	TYPE	TOTAL	TIME	TYPE	TOTAL	TIME	TYPE	TOTAL	TIME	TYPE	TOTAL		
								source is better than the other.	fat mass.<br><br>Tailor protein intakes to type of sport and exercise intensity.<br><br>Consume an extra amount of protein of approx. 0.25 - 0.3 g/kg body weight/day, equivalent to 15 - 25g.<br><br>No need to consume more than 1.6g/kg BW/day.<br><br>Tailor protein intake to match the length of training.								
Abreu et al. (2021)	-	-	During offseason, adjust total calorie intake to prevent weight gain.<br><br>Muscle protein synthesis can represent approx. 500 kcal/day.	-	-	-	Consume meals with essential and non-essential amino acids after matches.<br><br>Anabolic 'window of opportunity' might not exist. Consume quality protein with all amino acids, up to 2-4 hours after a match.	Consume quality protein with all the amino acids up to 2-4 hours after a match.	During the season and offseason, aim for a protein intake of 1.6-2.0g/kg/day, depending on body composition goals.<br><br>20 - 40g protein.	-	-	-	-	-	-	-	-
Collins et al. (2021)	-	-	Manipulate energy intake to elicit changes in fat mass or skeletal muscle mass.	-	-	Daily carbohydrate intake of 4-8 g/kg body mass supports preseason training that aims to manipulate body composition (weight gain and lean mass gain).	-	-	-	-	-	-	-	-	-	Creatine recommended.<br>Initial loading phase: 20g/day in 4 equal doses for 5-7 days<br>Followed by maintenance phase of 3-5g/day in a single dose<br>Alternative approach: 2-5g/day for 28 days<br>Co-ingestion with protein/carbohydrate mixture (approx. 50g protein)	-
Ferrando et al. (2023)	-	-	-	-	-	-	-	-	-	-	-	-	-	-	-	Free-form EAA supplementation maybe recommended.<br><br>Consider using free-form EAAs over intact protein (whey) for ease of consumption close to before and during exercise.<br><br>Consume a combination of all EAAs and in dosages ranging from 1.5 g to 18 g. Consume a greater percentage of leucine (%/g) in compositions of EAAs to maximally stimulate muscle protein synthesis populations that demonstrate anabolic resistance (aging).<br><br>Combine the ingestion of EAA with either resistance or aerobic exercise, especially in the aging population.	-
Sims et al. (2023)	-	-	An EA > 45 kcal/ kg FFM/ day provides enough energy for weight gain and muscle hypertrophy.	-	-	-	The consumption of protein post-exercise is	-	In some populations as much as a 60%	-	-	-	-	-	-	Creatine supplementation for women is recommended.	-

STUDY	CALORIES			CARBOHYDRATE			PROTEIN			FAT			MICRONUTRIENTS			SUPPLEMENTS	FLUID
	TIME	TYPE	TOTAL	TIME	TYPE	TOTAL	TIME	TYPE	TOTAL	TIME	TYPE	TOTAL	TIME	TYPE	TOTAL		
							necessary to maximize the stimulation of skeletal muscle hypertrophy.		increase from habitual protein intake may be needed to support this goal.							Recommended dosage of a loading dose of ~ 20 g per day for 5 days (4- 5 g doses taken every 4 hours), followed by 3-5 g per day.	
Leaf et al. (2024)	-	-	-	-	-	For body composition, ketogenic diets are not recommended for increasing fat-free mass.	-	-	-	-	-	-	-	-	-	-	-
Number of papers (total 73)	5	3	28	5	0	13	33	22	36	0	0	1	0	0	2	29	2
