# Supplementary material for: Dietary Recommendations for Body Mass and Composition Manipulation in Male and Female Athletes: a Scoping Review of Consensus Statements, Position Stands and Practice Guidelines from International Expert Groups
Source: Sports Med. 2025 Aug 21;55(10):2445–87. doi: 10.1007/s40279-025-02285-4 (PMC12513969; doi:10.1007/s40279-025-02285-4)
Supplement: Supplementary file 4 — Supplementary file4 (PDF 544 kb) [file 40279_2025_2285_MOESM4_ESM.pdf]

Dietary recommendations for body mass and composition manipulation in male and female athletes: A scoping review of consensus statements, position stands and practice guidelines from international expert groups

Lauren V. Delany[1,2], Nessan Costello[1], Ben Jones[1,3,4,5], Susan H. Backhouse[1]

[1] Carnegie School of Sport, Leeds Beckett University, Leeds, United Kingdom

[2] Sale Sharks Rugby Club, Manchester, United Kingdom

[3] England Performance Unit, Rugby Football League, Manchester, United Kingdom

[4] Division of Physiological Sciences, Department of Human Biology, Faculty of Health Sciences, The University of Cape Town and the Sports Science Institute of South Africa, Cape Town, South Africa

[5] Premiership Rugby Limited, London, United Kingdom

Corresponding author:

Lauren Delany

Carnegie School of Sport, Leeds Beckett University, Headingly Campus, Leeds, United Kingdom, LS6 3QU

l.delany@leedsbeckett.ac.uk

Supplementary Information 4: Diet and supplement recommendations to decrease athlete body mass or fat mass

STUDY	CALORIES			CARBOHYDRATES			PROTEIN			FATS			MICRONUTRIENTS			SUPPLEMENTS	FLUID
	TIMING	TYPE	TOTAL	TIMING	TYPE	TOTAL	TIMING	TYPE	TOTAL	TIMING	TYPE	TOTAL	TIME	TYPE	TOTAL		
American College of Sports Medicine (1976)	-	-	Aim to meet the daily calorie requirements from a balanced diet.<br><br>Factors influencing energy needs include age, body surface area, growth, and physical activity levels.<br><br>Minimum calorie needs of wrestlers' ranges from 1200 to 2400 kcal/day.	-	-	-	-	-	-	-	-	-	-	-	-	-	Discourage the practice of fluid deprivation and dehydration.
Burke (1995)	-	Focus on nutritious foods as athletes will need to meet nutrient needs from less kilojoules.<br><br>Be prudent with alcohol as it is low in nutrients is often associated with unwise eating.	Avoid chronically low energy intakes health problems and inadequate nutrient intake.<br><br>Reduce your typical energy intake by e.g. 500-1000 kcal/ day (2.1-4.2 MJ/ day) to lose body fat (ensures adequate food and nutrient intake).<br><br>Do not decrease your intake below 1200-1500 kcal/ day (5-6.4 MJ/ day).	-	Be prudent with sugar as it is low in nutrients.<br><br>Choose carbohydrate food staples with a high micronutrient density and a moderate fat content to support an increased micronutrient need and/or a restricted energy budget.<br><br>Make meals filling by choosing high-fibre forms of foods.	-	-	-	-	-	Choose low-fat versions of nutritious protein foods.<br><br>Minimize added fats and oils in cooking and food preparation.<br><br>Enjoy high-fat snack and sweet foods as occasional treats rather than everyday foods.	Reduce intake of fats and oils.	--		Consider calcium intake for those who follow:<br>- a restricted energy intake<br>- dietary extremism and fad diets<br>- vegan (complete vegetarian) eating patterns<br>- situations where athletes are (or consider themselves to be) intolerant to dairy products	Consider a broad-range, low-dose vitamin/mineral supplement if restricting intake below 1500 kcal/day (6.4 MJ/day) for prolonged periods.	-
Williams (1995)	-	-	-	-	-	-	-	-	-	-	-	-	-	-	-	Boron supplement not recommended.	-
Oppliger et al. (1996)	-	-	Emphasize the need for daily caloric intake obtained from a balanced diet<br><br>Daily calorie intake should be determined based on RDA guidelines and physical activity levels.<br><br>Minimal calorie needs for wrestlers, ranges from 1700 to 2500 kcal/d and rigorous training may increase the requirement up to an additional 1000 calories per day. (Allows for gradual weight loss).	-	-	Choose a balanced diet high in carbohydrates (>55% of calories)	-	-	Choose a balanced diet with adequate protein (15%-20% of calories, 1.0-1.5 g/kg body weight)	-	-	Aim for a balanced diet low in fat (<30% of calories)	-	-	-	-	-

STUDY	CALORIES			CARBOHYDRATES			PROTEIN			FATS			MICRONUTRIENTS			SUPPLEMENTS	FLUID
	TIMING	TYPE	TOTAL	TIMING	TYPE	TOTAL	TIMING	TYPE	TOTAL	TIMING	TYPE	TOTAL	TIME	TYPE	TOTAL		
Manore et al. (2000)	-	Reduce intake of energy-dense snacks.	Avoid low-energy diets or extreme energy restrictions as they will not sustain athletic training and may adversely influence an athlete's performance.<br><br>Aim to decrease normal energy intake by 10% to 20% to support weight loss without the athlete feeling deprived or overly hunger.<br><br>Specific recommendations for individual energy components should be based on body size, weight and body composition goals, the sport being performed, and sex of the athlete.	Consuming carbohydrates during exercise is even more important in situations when athletes restrict energy intake for weight loss.	Increase intake of whole grains and cereals, beans, and legumes.	Difficult to meet optimal carbohydrate targets (4 to 5 g/kg in a 60 kg athlete) with energy intakes below 2,000kcal/day and 60% intake from carbohydrates.<br><br>Typically, diets containing 20-25% energy from fat have been recommended to facilitate adequate carbohydrate intake and to assist in weight management where necessary.	-	Accordingly, use of low-fat dairy products and lean meats, fish, and poultry is suggested.	Dieting athletes should not have too little protein intakes.	-	Substitute lower-fat foods for whole-fat foods.	Decrease in energy intake of 10% to 20% of normal intake.<br><br>Consider reducing fat intake but acknowledge a lower-fat diet will not guarantee weight loss if a negative energy balance (reduced energy intake and increased energy expenditure) is not achieved.<br><br>Fat intake should not decrease below 15% of total energy intake, because some fat is essential for good health.<br><br>Typically, diets containing 20-25% energy from fat have been recommended to facilitate adequate carbohydrate intake and to assist in weight management where necessary.	-	Consider use of low-fat dairy products to support calcium intakes.<br><br>Aim for 5+ daily servings of fruits and vegetables to provide nutrients and fibre.	Consider intake in athletes at greatest risk of micronutrient deficiencies including those who:<br>- are dieting<br>- restrict energy intake<br>- use severe weight-loss practices<br>- eliminate one or more food groups from their diet.<br><br>Dieting athletes need to maintain adequate calcium intakes.	Athletes at the greatest risk of poor micronutrient status and may require a multivitamin/ mineral supplement include those who:<br>- are dieting<br>- restrict energy intake<br>- use severe weight-loss practices<br>- eliminate one or more foods or food groups from their diet<br>- are sick or recovering from injury<br>- has a specific micronutrient deficiency<br>- consume high-carbohydrate diets with low micronutrient density.	Dehydration as a means of reaching a body-weight goal is not recommended.
Loucks (2004)	-	-	Avoid low energy availability as the hazard in such sports.<br><br>A reduced energy intake may not be necessary if increasing lean body mass at the same time as decreasing fat mass.<br><br>Manage carbohydrate, protein and fat balance to achieve a sport-specific body size and body composition.	-	-	-	-	-	-	-	Induce a negative fat balance by minimising the intake of saturated fats.	To reduce fat mass, athletes need to induce negative fat balance.	-	-	-	-	-
Maughan et al. (2004)	-	-	-	-	-	-	-	-	-	-	-	-	-	-	-	Carnitine not recommended.<br><br>Strongly discourage use of any supplements that are prohibited by	-

STUDY	CALORIES			CARBOHYDRATES			PROTEIN			FATS			MICRONUTRIENTS			SUPPLEMENTS	FLUID
	TIMING	TYPE	TOTAL	TIMING	TYPE	TOTAL	TIMING	TYPE	TOTAL	TIMING	TYPE	TOTAL	TIME	TYPE	TOTAL		
																doping regulations, raise questions about safety or have significant health risks e.g. caffeine, ephedrine, or aspirin.	
Burke et al. (2006)	Aim for a negative energy balance across the day (through diet and training) or for substantial portions of the day to reduce body fat.	Avoid foods that are energy-dense but low in nutrient density. Instead choose foods that are high in nutrient density so that nutrient needs are meet from a lower intake of energy.<br><br>To manage hunger choose foods that are low in energy density or high in satiety value (e.g. low glycaemic index or protein-containing)	Consider, adjust and individualise energy intakes when manipulating muscle mass and body fat.<br><br>Aim for a negative energy balance.<br><br>Avoid a low availability of energy, (defined as total energy intake minus the energy cost of the athlete's exercise programme), as causes health problems. Aim for an energy availability of above 30 kcal (125 kJ) per kilogram of fat free mass to maintain normal menstrual function.<br><br>Females should avoid over-restricting energy intake to achieve loss of body fat as this can cause metabolic, reproductive, and bone disruptions.<br><br>Soccer players should not undertake a diet and exercise programme that allows or specifically promotes a substantial energy deficit.	-	-	A low-carbohydrate diet is not a suitable weight-loss programme for an active soccer player.<br><br>Avoid low carbohydrate availability, as this may underpin some of the metabolic disturbances seen in instances of low energy availability.<br><br>Aim to achieve a carbohydrate intake that meets the fuel requirements of their training programme and optimizes restoration of muscle glycogen stores between training sessions and before matches.<br><br>General carbohydrate recommendations should be fine-tuned with individual consideration of total energy needs, specific training needs, and feedback from training/match performance.<br><br>Moderate daily recovery and match preparation with periods of energy restriction for fat loss = 5-7g carbohydrate/ kg/ day.	-	-	To manage hunger, choose foods that are low in energy density or high in satiety value (e.g. low glycaemic index or protein-containing).	-	Moderate saturated fats.	Fat intake should be moderated.	-	-	-	-	-
Hespel et al. (2006)	-	-	Aim for an energy deficit through reducing energy intake, to reduce body weight.<br><br>Not all players can achieve the expected body fat goal in this way.	-	-	-	-	-	-	-	-	-	-	-	-	Strongly discourage use of any supplements at any level of competition that are on the list of banned substances issued by WADA, can cause a positive doping test and can be dangerous to health e.g.	-

STUDY	CALORIES			CARBOHYDRATES			PROTEIN			FATS			MICRONUTRIENTS			SUPPLEMENTS	FLUID
	TIMING	TYPE	TOTAL	TIMING	TYPE	TOTAL	TIMING	TYPE	TOTAL	TIMING	TYPE	TOTAL	TIME	TYPE	TOTAL		
																ephedrine and ephedra.	
Burke et al. (2007)	-	-	Do not practice extreme levels of energy restriction to achieve loss of body weight/body fat, without considering macro- and micro-nutrient intake.<br><br>Avoid inadequate energy intakes as this can impair hormonal balance, bone health, and the immune system.	-	-	The targets of 7 - 10g/kg/day for high volume training and 5 - 7g/kg/day for more moderate exercise loads. These should be fine-tuned according to overall nutritional goals and performance feedback from each athlete.<br><br>Such recommendations may be unfeasible for runners, particularly females, whose focus on low body mass and percent body fat requires energy restriction and, by association, a lower carbohydrate intake.<br><br>Aim to periodize nutrition goals and dietary carbohydrates intakes over the season.<br><br>Lower carbohydrate intakes and physique goals are the priority during training periods.	-	-	Estimated protein requirements are nearly twice those of sedentary individuals, 1.6-1.7 g/ kg/ day.<br><br>No research in female athletes but nitrogen balance data imply that the requirements for women are about 25% lower than those for men - that is, 1.2 - 1.3g/ kg/ day.<br><br>Most athletes will achieve these protein intakes from an everyday diet providing 10 - 15% of energy as protein and adequate energy.<br>Use grams per kilogram vs percentage of the diet to avoid low intakes that can be seen in energy restricting athletes.<br><br>A low energy intake will also have a negative effect on protein requirements.	-	-	-	-	-	-	-	-
Houtkooper et al. (2007)	-	-	-	-	-	-	-	-	-	-	-	-	-	-	-	Consider a low-dose vitamin and mineral supplement, that does not exceed the daily recommended intakes to meet nutrient needs in those who:<br>- have diets that are restrictive in total energy<br>- have diets that are limited in food variety<br>- severely restrict or eliminate specific food groups (i.e. meat or dairy)	-
Manore et al. (2007)	-	-	Do not reduce energy availability below 30 kcal/ kg FFM/ day, as this impairs reproductive and skeletal health in	-	-	-	-	-	-	-	-	-	-	-	-	-	-

STUDY	CALORIES			CARBOHYDRATES			PROTEIN			FATS			MICRONUTRIENTS			SUPPLEMENTS	FLUID
	TIMING	TYPE	TOTAL	TIMING	TYPE	TOTAL	TIMING	TYPE	TOTAL	TIMING	TYPE	TOTAL	TIME	TYPE	TOTAL		
			both males and females within 5 days.<br><br>Athletes should aim to maintain energy availability between 30 and 45 kcal/ kg FFM/ day (125 - 188 kJ/ kg FFM/ day) for weight loss. Weight loss example: body weight 61.5kg, body fat 13.5%, FFM 53.2kg, EI 2382 kcal/day, EEE 520 kcal/day, EA 35 kcal/kg FFM/day.														
Maughan et al. (2007)	-	-	-	-	-	-	-	-	-	-	-	-	-	-	-	Consider a broad-spectrum, low-dose multivitamin and mineral supplement if restricting energy intake to help reduce body fat.	-
O'Connor et al. (2007)	-	Design a diet with lower energy density (less energy/g food). Energy dense diets promote 'passive over consumption'.	Avoid insufficient energy availability when planning to restrict energy intake.<br><br>Aim to gradually use a modest energy and fat restriction to reduce weight or fat, and this will ensure carbohydrate and nutrient needs are satisfied.<br><br>Start with an energy deficit of 2100 kJ (500 kcal/d) from theoretical needs.<br><br>Energy availability must not fall below 125 kJ or 30 kcal/kg/d fat free mass.	-	A low GI diet for improving satiety and weight management in athletes is not recommended.	Assist athletes to understand their own specific energy and carbohydrate needs.<br><br>Avoid advice to eat a 'high', unlimited carbohydrate diet may result in over-consumption.<br><br>Use modest energy and fat restriction to ensure carbohydrate and nutrient needs are satisfied.<br><br>Low carbohydrate diets (< 5 g/k/d) for weight/fat loss are not recommended.	-	-	-	-	-	Athletes seeking to reduce weight or fat are recommended to do so gradually using modest energy and fat restriction.<br><br>Ad libitum reduction in fat intake to create a modest decrease in both energy intake and weight/fat loss can be appropriate for athletes with high energy expenditures and modest reduction goals.<br><br>Ad libitum reduction in fat intake as a method, is less restrictive, avoids jeopardising carbohydrate intake and may be less likely to result in binge eating.	-	Adequate intake of dairy foods may play a positive role in weight/fat loss.	-	No dietary supplement is recognized as providing significant, effective support to enhance weight or fat loss in athletes.<br><br>Planning pre- and post- training nutritional support around meals can allow for nutrient and recovery needs can be managed without the use of energy dense supplements.<br><br>Insufficient evidence to support use of L-carnitine, chromium picolinate, hydroxy-methyl butyrate (HMB) in weight management.<br><br>Use of pharmacological agents for physique management, weight or fat loss, is not recommended due to bans by sports drug agencies and health and ethical reasons.	-

STUDY	CALORIES			CARBOHYDRATES			PROTEIN			FATS			MICRONUTRIENTS			SUPPLEMENTS	FLUID
	TIMING	TYPE	TOTAL	TIMING	TYPE	TOTAL	TIMING	TYPE	TOTAL	TIMING	TYPE	TOTAL	TIME	TYPE	TOTAL		
Stellingwerff et al. (2007)	-	-	-	-	-	In general prep and specific prep phases, carbohydrate intake should be high, ranging from 7 to 10g CHO/kg BW/day.	-	-	General prep and specific prep phases: Protein recommendations are 1.5 - 1.7 g PRO / kg body weight/ day.	-	-	General prep phase: Recommended fat intake is highest during this phase (1.5 - 2.0 g fat/ kg BW/day), translating into approx. 30% of total energy.<br><br>Specific preparation phase:<br>Due to the decreased total energy expenditure, reduce dietary fat intake to about 20 - 25% of total energy, or 1 - 1.5 g fat/ kg BW/ day.	-	-	-	-	-
Bonci et al. (2008)	-	-	Avoid prolonged energy and nutrient deprivation.	-	-	-	-	-	-	-	-	-	-	-	-	-	-
Kerksick et al. (2008)	-	-	-	-	-	-	-	Regularly consume various protein sources with carbohydrate and this may favourably impact body composition.	-	-	-	-	-	-	-	-	-
New Zealand Dietetic Association (2008)	-	-	Aim to reduce the total energy intake while maintaining a balanced diet to lose weight/fat. However, ensure sufficient energy to enable the athlete to train.<br><br>Aim for an energy deficit of 2100-4200 kJ/day to safely lose weight (0.5-1.0 kg/week), without compromising training and adequacy of nutrient intake.	-	-	-	-	-	-	-	-	-	-	-	In female athletes who limit energy to control body weight, consider intakes of calcium, iron, and zinc.	Consider a short-term course of a multivitamin or mineral supplement to reduce risk of micronutrient deficiencies, in those who:<br>- adopt fad diets<br>- unnecessarily restrict energy<br>- avoid one or more of the main food groups in their diet.	-
Rodriguez et al. (2009)	-	Reduce intake of energy dense snacks.	Females should avoid an energy availability below 30 kcal/kg FFM.<br><br>Aim to decrease energy intake by 10% to 20% of normal intake for weight loss, without the athlete feeling deprived or overly hungry.	-	Increase intake of whole grains and cereals, and legumes.	-	-	Choose the following protein sources: low-fat dairy products and lean meats, fish, and poultry.	Dieting athletes should not have too little protein intakes.	-	Decrease in energy intake of 10% to 20% of normal intake by substituting lower-fat foods for whole-fat foods.<br><br>Choose low-fat dairy products and lean meats.	Consider reducing fat intake but acknowledge a lower-fat diet will not guarantee weight loss if a negative energy balance (reduced energy intake and increased energy expenditure) is not achieved.<br><br>Fat intake	-	Consider use of low-fat dairy products to support calcium intakes.<br><br>Aim for 5+ daily servings of fruits and vegetables provide nutrients and fibre.<br><br>Ensure sufficient intake of	Consider intakes in athletes at greatest risk of micronutrient deficiencies including those who:<br>- restrict energy intake<br>- use severe weight-loss practices<br>- eliminate one or more food groups from their diet.<br><br>Athletes should consume diets	Consider a daily multivitamin and mineral supplement for those who:<br>- are dieting<br>- habitually eliminating foods or food groups<br>- are ill or recovering from injury<br>- have a specific micronutrient deficiency.	Dehydration as a means of reaching a body weight goal is contraindicated.

STUDY	CALORIES			CARBOHYDRATES			PROTEIN			FATS			MICRONUTRIENTS			SUPPLEMENTS	FLUID
	TIMING	TYPE	TOTAL	TIMING	TYPE	TOTAL	TIMING	TYPE	TOTAL	TIMING	TYPE	TOTAL	TIME	TYPE	TOTAL		
												should not decrease below 15% of total energy intake, because some fat is essential for good health.<br><br>Athletes who follow a low-fat diet are at greatest risk for poor antioxidant intakes.		fruits, vegetables and wholegrains. Limiting dietary intakes puts athletes at greatest risk for poor antioxidant intakes.	that provide at least the recommended dietary allowance (RDA) for all micronutrients.<br><br>Dieting athletes need to maintain adequate calcium intakes.<br><br>Consider intakes in athletes at greatest risk for poor antioxidant intakes including those following:<br>- a low-fat diet<br>- restricting energy intakes<br>- limiting dietary intakes of fruits, vegetables, and whole grains.		
La Bounty et al. (2011)	Aim to increase meal frequency as this can support the suppression of lean body mass losses during a hypocaloric diet; significant increases in lean body mass and anaerobic power and significant increases in fat loss.	-	Consider energy intake and expenditure when optimising body composition.	-	-	-	-	-	-	-	-	-	-	-	-	-	-
Loucks et al. (2011)	-	-	Aim for a diet and exercise plan that provides energy availability of 30-45 kcal/ kg FFM/ day while training to reduce body size or fatness.	-	-	-	-	-	-	-	-	-	-	-	-	-	-
Meyer et al. (2011)	-	-	-	-	-	-	-	-	When winter sport athletes' training intensity and/or volume increases and energy is restricted for weight loss aim for an adequate dietary protein intake of approx. 1.4-1.7g/ kg/ day.<br><br>When exposed to environmental extremes, winter sport athletes who restrict energy intake may benefit from additional protein to preserve lean tissue.	-	-	-	-	-	-	-	-
Phillips et al. (2011)	-	-	Aim for a relative energy deficit created by dietary	-	-	Reducing the intake of dietary carbohydrates is a	-	-	Depending on the caloric deficit, aim for an increased protein	-	-	-	-	-	-	-	-

STUDY	CALORIES			CARBOHYDRATES			PROTEIN			FATS			MICRONUTRIENTS			SUPPLEMENTS	FLUID
	TIMING	TYPE	TOTAL	TIMING	TYPE	TOTAL	TIMING	TYPE	TOTAL	TIMING	TYPE	TOTAL	TIME	TYPE	TOTAL		
			energy restriction and/or increased energy expenditure, for weight loss.			critically important step in promoting both greater weight loss and greater loss of body fat.<br><br>Consider also choosing a globally lower the glycaemic load of the diet by selecting low glycaemic-index (GI) carbohydrate sources.<br><br>A low carbohydrate, lower GI diets may be a problem for endurance athletes seeking to compete, as recommendations might not be met.<br><br>To optimize the ratio of fat-to-lean tissue mass loss during hypo energetic periods, athletes should lower their carbohydrate intake to approx. 40% of their energy intake (consume lower GI carbohydrates), equivalent to no more than 3-4 g/ kg/ day.<br><br>Consideration of how low carbohydrate intake should go, would be dictated by how much exercise performance may be compromised by consuming lower than recommended carbohydrates.			intake of 1.8-2.0 g/ kg/ day. This may help lean mass retention during periods of energy restriction to promote weight/fat loss.<br><br>Increase protein intake to approx. 20-30% of their energy intake or approx. 1.8-2.7 g/ kg/ day.								
Slater et al. (2011)	-	-	Avoid energy availability below the threshold of approx. 30 kcal/ kg fat free mass/ day as this is required to maintain normal endocrine regulation of the menstrual cycle.	-	Consider the strategic use of acute weight-loss strategies in the final 24-48 h before weigh-in for example a low-residue, low-volume meal plan.<br>This in combination with other strategies can induce a 2-3% body mass loss without promoting the health risks	-	-	-	Consider a relative increase in dietary protein intake to maintain lean body mass during weight loss.	-	-	-	-	-	-	-	Consider the strategic use of acute weight-loss strategies in the final 24-48 h before weigh-in for example moderation of fluid intake.<br>This in combination with other strategies, can induce a 2-3% body mass loss without promoting the health risks associated with

STUDY	CALORIES			CARBOHYDRATES			PROTEIN			FATS			MICRONUTRIENTS			SUPPLEMENTS	FLUID
	TIMING	TYPE	TOTAL	TIMING	TYPE	TOTAL	TIMING	TYPE	TOTAL	TIMING	TYPE	TOTAL	TIME	TYPE	TOTAL		
					associated with other acute weight-loss strategies.												other acute weight-loss strategies.
Stellingwerff et al. (2011)	-	-	Aim for a combined approach of slightly decreasing energy intake (approx. 500 kcal), while either maintaining or slightly increasing energy expenditure, to optimize body composition over an approximately 3-6 week period prior to the targeted competitive season.	-	-	-	-	-	During periods of negative energy balance in elite athletes aspiring to lose weight, consider increasing daily protein intake to approx. 35% of daily needs, or about 1.5-2.5 g/ kg BW/ day, which may help in lean tissue preservation.	-	-	-	-	-	-	-	-
Sundgot-Borgen et al. (2011)	-	-	No general guidelines for energy intake by athletes exist. Minimum intakes corresponding to 45 and 50 kcal/ kg body mass for females and males, respectively, have been suggested for athletes who exercise 490 min/ day.<br><br>Healthy dieting includes lowering energy intake by a modest amount per day to achieve gradual weight loss.<br><br>Aim to consume sufficient energy to avoid menstrual irregularities.<br><br>Aim for an energy deficit of approx. 500 kcal/ day to induce a weight loss of 0.5 kg per week, but there are individual differences. This can be achieved by reducing energy intake, increasing energy expenditure, or a combination of the two.	-	-	The diet plan should aim at a carbohydrate intake corresponding to 4-6 g/ kg.	Emphasise recovery meals containing carbohydrates and protein within 30 minutes after training to optimize recovery.	-	Aim for a protein intake of 1.4-2.0 g/ kg.	-	-	The diet plan should aim at 15-20% fat.	-	Include dairy food sources to meet the recommended dietary intake of calcium.	--	Take a multi-vitamin/mineral supplement and omega-3 fatty acids during a period of weight loss to ensure sufficient micronutrient intake and essential fats.	-
Turocy et al. (2011)	-	-	Total caloric intake should be determined by calculating the basal metabolic rate (BMR) and the energy needs for activity.	-	-	Carbohydrates should provide 55% to 70% of the total caloric need of athletes and active people.<br><br>Needs may be as high as 12 g or	-	-	-	-	-	-	-	-	A healthy diet or meal plan should supply essential nutrients.<br><br>Appropriate intake of non-energy-producing essential	Be cautious when ingesting ergogenic and dietary aids.<br>Take supplements under the advisement of those knowledgeable of	Appropriate intake of non-energy-producing essential nutrients (e.g., water) is needed to facilitate energy

STUDY	CALORIES			CARBOHYDRATES			PROTEIN			FATS			MICRONUTRIENTS			SUPPLEMENTS	FLUID
	TIMING	TYPE	TOTAL	TIMING	TYPE	TOTAL	TIMING	TYPE	TOTAL	TIMING	TYPE	TOTAL	TIME	TYPE	TOTAL		
			Table 4. Determining Total Caloric Needs: Calculations included<br>Table 5. Determining Energy-Producing Nutrient Intake: Calculations included<br><br>Aim for a healthy diet or meal plan that provides adequate calories to achieve body weight and composition goals.<br><br>Avoid a calorie intake that is too low or too high to support the desired lean body mass as this will negatively affect metabolic function and body composition.<br><br>Use the U.S. Department of Agriculture's Food Pyramid Guide can be used to ensure adequate nutrient intake.<br><br>The AT or other trained health care professional can also use the appropriate Food Guide Pyramid to calculate the recommended caloric intake level based on the individual's goal weight.<br><br>One pound (0.5 kg) of fat is equal to 3500 kilocalories of energy; therefore, increases or decreases in calories to the level needed to maintain ideal lean mass will help to achieve body fat goals.<br><br>Caloric and nutrient intake should be based on lean body mass, desired body composition, goal weight, and sport or activity requirements.			more/kg body weight.									nutrients (e.g., vitamins, minerals, water) is needed to facilitate energy creation and maintain other body processes.	the requirements of sports and other governing organizations.	creation and maintain other body processes.

STUDY	CALORIES			CARBOHYDRATES			PROTEIN			FATS			MICRONUTRIENTS			SUPPLEMENTS	FLUID
	TIMING	TYPE	TOTAL	TIMING	TYPE	TOTAL	TIMING	TYPE	TOTAL	TIMING	TYPE	TOTAL	TIME	TYPE	TOTAL		
Sundgot-Borgen et al. (2013)	To avoid extra energy by adding a recovery meal, have a recovery meal as one of the planned meals for the day.	Consume foods with a low energy density but high nutrient density to provide satiety.	Female athletes should consume sufficient energy to avoid menstrual irregularities (>30–45 kcal/kg/day).<br><br>To induce a weight loss of 0.5 kg/week an energy deficit of about 500 kcal/day is needed, but there will be individual differences in how this is achieved.<br><br>Depending on the training phase, this energy deficit is most likely induced by a combination of decreased energy intake and increased energy expenditure.<br><br>Smaller, perhaps intermittent, deficits are still possible but monitoring of performance, immune and psychological parameters is essential.	Recovery meals containing carbohydrates and protein should be encouraged within 30 min after training sessions to optimise recovery.	-	Daily meal plans should contain at least 3–5 g/kg of carbohydrate, although this regime depends on the sport (eg, endurance athletes need more carbohydrate).<br><br>Recovery meals should contain carbohydrates at approximately 1 g/kg and these should include a variety of foods.	Recovery meals containing carbohydrates and protein should be encouraged within 30 min after training sessions to optimise recovery.	-	Daily meal plans should contain 1.5–2 g/kg of protein, although this regime depends on the sport (eg, endurance athletes need more carbohydrate).<br><br>Recovery meals should contain protein at approx. 20–25 g or 0.25 g/kg and these should include a variety of foods.<br><br>To maintain muscle mass during phases of energy deficits, extra protein, possibly in combination with strength training, is recommended.	-	-	Daily meal plans should contain ≥15–20% of total energy intake from fat, although this regime depends on the sport (eg, endurance athletes need more carbohydrate).	-	-	If blood tests indicate specific micronutrient issues (eg, iron, vitamin B12 or vitamin D deficiencies) the athlete should make appropriate adjustments to the diet, or use safe and effective supplementation, and monitoring.	Consider a multivitamin/ mineral supplement and ω-3 fatty acids during weight-loss interventions to assure sufficient micronutrient and essential fat intake.<br><br>Consider safe and effective supplementation for any micronutrient deficiencies highlighted from blood tests.	-
Team Physicians (2013)	Spread calorie intake throughout the day to maintain lean body mass and reduce body fat.	Substitute high calorie foods for low calorie foods.	Create a negative energy balance by adjusting energy intake. Aim for increases in energy expenditure and moderate decreases in energy intake of 250-1000 calories per day (leads to approximately 0.5-2.0 lb/week).<br><br>Avoid an excessive energy restriction which can cause adverse health effects. Minimum intakes for female athletes 1200-1400 calories per day and males 1500-1700 calories per day.	-	-	-	-	-	-	-	-	-	-	-	An athlete cannot maintain adequate micronutrient levels with these restrictive amounts of energy intake.	-	Athletes should remain hydrated during weight loss.
Wilson et al. (2013)	-	-	-	-	-	-	-	-	-	-	-	-	-	-	-	HMB ingestion in conjunction with a structured exercise program may result in greater declines in fat mass (FM).<br>In individuals who are moderately calorically	-

STUDY	CALORIES			CARBOHYDRATES			PROTEIN			FATS			MICRONUTRIENTS			SUPPLEMENTS	FLUID
	TIMING	TYPE	TOTAL	TIMING	TYPE	TOTAL	TIMING	TYPE	TOTAL	TIMING	TYPE	TOTAL	TIME	TYPE	TOTAL		
																restricted may improve fat loss and prevent declines in LBM by supplementing with HMB.<br><br>HMB alone can decrease body fat and increase skeletal muscle mass and strength in master's level athletes or aging populations.<br><br>HMB's acute effects likely depend upon supplementation pre-exercise.<br>To optimize HMB's chronic effects, consume 3 g daily, divided into three equal servings. A minimum of two weeks prior to a potentially damaging skeletal muscle event.<br>If taking HMB-Ca, consume 3 g, at least 60 minutes prior to intense exercise.<br>If consumed with glucose it may need to be taken as long as two hours prior to training.<br>If taking HMB-FA consume 30-60 minutes prior to exercise.	
Benardot et al. (2014)	-	-	Reduce energy availability through dietary restriction.<br><br>Avoid a low energy availability (energy consumed minus exercise energy expended in training) as this will cause health complications.<br>Avoid an energy intake below 20-30 kcal/kg lean body mass/day.	-	-	-	Consider eating frequency as this influences the total daily protein intakes recommended.	Recommended protein intake can generally be met through diet alone.<br><br>The use of protein drinks (i.e., whey protein isolate) and related packaged foods may assist in the timely intake of protein immediately after training or competition.<br><br>Protein quality influences the total daily protein intakes recommended.	Slightly higher protein intake may be required for athletes striving to lose (fat) weight to better maintain lean muscle mass.<br><br>Total daily protein intake to optimize the adaptation from daily training and promote recovery after exercise is approximately 1.2-1.7 g/kg body mass.	-	-	-	-	-	Consider intakes in divers at greatest risk of micronutrient deficiencies including those who:<br>- restrict energy intake<br>- use severe weight-loss practices<br>- eliminate one or more food groups from their diet<br>- consume high- or low-carbohydrate diets of low micronutrient density.<br><br>Divers should consume diets that provide at least the Dietary Reference Intakes for all micronutrients.	The use of protein drinks (i.e., whey protein isolate) and related packaged foods may assist in the timely intake of protein immediately after training or competition.	-

STUDY	CALORIES			CARBOHYDRATES			PROTEIN			FATS			MICRONUTRIENTS			SUPPLEMENTS	FLUID
	TIMING	TYPE	TOTAL	TIMING	TYPE	TOTAL	TIMING	TYPE	TOTAL	TIMING	TYPE	TOTAL	TIME	TYPE	TOTAL		
Cox et al. (2014)	-	-	-	-	-	Water polo daily requirements of carbohydrates are likely within 4–8g/kg body mass/day.<br><br>The lower range recommendations for carbohydrate intake are probably suitable for athletes with high body fat levels (given that recommendations are expressed relative to body mass), or female water polo players striving to reduce body fat levels.	-	-	-	-	-	-	-	-	-	-	-
Derave et al. (2014)	-	-	-	-	-	-	-	Consider protein supplements as a guaranteed amount of high-quality protein without additional energy, which can be useful for the swimmer with a restricted energy budget.	-	-	-	-	-	-	Micronutrient deficiencies are likely when food intake is restricted to achieve physique goals or for other reasons.	Individualise protein intake from normal foods or supplements. Protein supplements provide a guaranteed amount of high-quality protein without additional energy, which can be useful on a restricted energy budget, e.g. whey protein.	-
Melin et al. (2014)	-	-	Avoid persistent restricted energy intake and low energy availability (EA) as this can negatively impact the function of many body systems in both female and male athletes.<br><br>It is ok to engage in healthy periodic dieting or occasional use of more extreme weight loss methods, such as short-term restrictive diets with low EA (< 125 kJ/kg of fat-free mass, or FFM, per day, or 30 kcal/kg of FFM/day).	-	-	-	-	-	-	-	-	-	-	-	-	-	-
Mountjoy et al. (2014)	-	-	Healthy dieting can include the occasional use of more extreme weight loss methods such as short-term restrictive diets of <30 kcal/kg FFM/day.	-	-	-	-	-	-	-	-	-	-	-	-	-	-

STUDY	CALORIES			CARBOHYDRATES			PROTEIN			FATS			MICRONUTRIENTS			SUPPLEMENTS	FLUID
	TIMING	TYPE	TOTAL	TIMING	TYPE	TOTAL	TIMING	TYPE	TOTAL	TIMING	TYPE	TOTAL	TIME	TYPE	TOTAL		
Mujika et al. (2014)	-	-	Aim to reduce energy intake minimally, within the temporary constraints for the mass loss (i.e., goal date by which the mass must come off).<br><br>Use an individualized approach to fuel needs based on each athlete's body size and composition and each athlete's specific training and competition program.<br><br>Restrict energy intake during base training phases of swimmers aiming to reduce excess body fat.<br><br>Periodize calories and macronutrient intake towards desired body composition change.	-	-	Guidelines for nutrition should have an individualized approach to fuel needs based on each aquatic athlete's body size and composition and each athlete's specific training and competition program.<br><br>For instance, energy and carbohydrate intake could be restricted during base training phases of swimmers aiming to reduce excess body fat.	-	-	Consider a high protein diet for aquatic sport athletes desiring to maintain muscle while losing total body mass.	-	-	-	-	-	-	-	-
Robertson et al. (2014)	-	-	-	-	-	Recommended carbohydrate intakes 5 - 7g /kg /day. Adjust carbohydrate targets depending on body composition goals.	-	-	-	-	-	-	-	-	-	-	-
Shaw et al. (2014)	-	-	Adapt total energy intake according to physique manipulation goals.	-	-	Adapt carbohydrate intake according to physique manipulation goals.	-	-	-	-	-	-	-	-	-	-	-
Thomas et al (2016)	-	Do not misuse alcohol as this can compromise the management of body composition.	Aim for an appropriate energy intake as this assists in manipulating body composition.<br><br>Avoid continuous dieting and chronic periods of low EA through insufficient energy intake for weight loss.<br><br>Aim for a slight energy deficit to achieve a slow rather than rapid rate of loss, for example ~250-500 kcal/d from their periodized energy needs, while either maintaining or slightly increasing energy	-	-	Carbohydrate recommendations are 3-10 g/kg BW/day and up to 12 g/kg BW/d for extreme and prolonged activities. Adapt based on body composition goals.	-	-	When energy availability is reduced (e.g., to reduce body weight/fat), higher protein intakes are needed to support MPS and retention of fat-free mass.<br><br>Dietary protein intake to support adaptations ranges from 1.2 to 2.0 g/kg/d.<br><br>In cases of energy restriction, elevated protein intakes as high as 2.0 g/kg/day or higher when spread over the day may be advantageous in preventing fat-free mass loss.<br><br>Higher doses (i.e., >40 g dietary protein) may only be prudent for	-	-	Individualize fat intake based on body composition goals.<br><br>Avoid excessively restricting fat intake to lose body weight or improve body composition.<br><br>Avoid chronically low-fat diets below 20% of energy intake since the reduction in dietary variety is likely to reduce the intake of nutrients such as fat-soluble	-	-	Athletes should consume diets that provide at least the Recommended Dietary Allowance (RDA)/Adequate Intake (AI) for all micronutrients.<br><br>Consider intakes in athletes at greatest risk of micronutrient deficiencies including those who:<br>- restrict energy intake<br>- use severe weight-loss practices<br>- eliminate complete food groups from their diet	Consider micronutrient supplementation for those who may have sub-optimal intake through:<br>- frequently restricting energy intake<br>- relying on extreme weight-loss practices<br>- eliminating one or more food groups from their diet<br>- consume poorly chosen diets.<br><br>Particular nutrients which may be sub-optimal include calcium, vitamin D, iron, and some antioxidants.	-

STUDY	CALORIES			CARBOHYDRATES			PROTEIN			FATS			MICRONUTRIENTS			SUPPLEMENTS	FLUID
	TIMING	TYPE	TOTAL	TIMING	TYPE	TOTAL	TIMING	TYPE	TOTAL	TIMING	TYPE	TOTAL	TIME	TYPE	TOTAL		
			expenditure over approximately 3-6 weeks.						the largest athletes, or during weight loss.			vitamins and essential fatty acids, especially n-3 fatty acids.			- follow other extreme dietary philosophies.		
Aragon et al. (2017)	No advantage of intermittent caloric restriction over daily caloric restriction to improving body composition.	-	Aim for a sustained calorie deficit.<br><br>The higher the baseline body fat level, the more aggressively the calorie deficit may be imposed.<br><br>This net hypocaloric balance can either be imposed linearly/daily, or non-linearly over the course of the week, determined by individual preference, tolerance, and athletic goals.	-	-	-	-	-	Consider increasing dietary protein beyond current recommendations for athletic populations may improve body composition. Protein requirements in athletic populations is 1.4-2.0g/kg/day.<br><br>Higher protein intakes (2.3-3.1 g/kg FFM) may be required to maximise muscle retention in lean, resistance-trained subjects in hypocaloric conditions.<br><br>Adequate protein, resistance training, and an appropriate rate of weight loss should be the primary focus for achieving the objective of LM retention (or gain) during FM loss.	-	-	-	-	-	-	-	-
Jager et al. (2017)	When restricting calorie intake, aim to consume three to four whole meals.	-	Aim for a hypo energetic diet by reducing energy intake by 30-40% and increasing daily protein to maximize the loss of fat tissue while also promoting the maintenance of fat-free mass.	-	-	-	Individuals attempting to restrict caloric intake should consume three to four whole meals consisting of 20-40 g of protein per meal.	-	Higher protein intakes (>3.0 g/kg/d) may promote loss of fat mass in resistance-trained individuals.<br><br>To maximize the loss of fat tissue while promoting the maintenance/increase of fat-free mass, increase daily protein intake through a combination of food and supplementation to levels above the recommended daily allowance (RDA 0.8 g/kg/day) to 1.2-2.4 g/kg/day for the endurance and strength/power athletes) and restrict energy intake (30-40% reduction in energy intake).<br><br>20-40 g of protein per meal.	-	-	-	-	-	-	-	-
Kerksick et al. (2017)	Aim to evenly spreading out calories across the day and avoid extended periods of time where no food is consumed.	-	-	-	-	-	Avoid extended periods of time where no food, protein in particular, is consumed.<br><br>Pre- or post-exercise nutrition interventions of protein alone or with	-	-	-	-	-	-	-	-	-	-

STUDY	CALORIES			CARBOHYDRATES			PROTEIN			FATS			MICRONUTRIENTS			SUPPLEMENTS	FLUID
	TIMING	TYPE	TOTAL	TIMING	TYPE	TOTAL	TIMING	TYPE	TOTAL	TIMING	TYPE	TOTAL	TIME	TYPE	TOTAL		
	Consume a greater proportion of calories during breakfast alongside protein, may improve weight loss, body composition changes, and health-related markers.						carbohydrate, may improve body composition. The size and timing of pre-exercise meal may impact the need for post-exercise protein intake.										
Maughan 2018	-	-	-	-	-	-	-	-	-	-	-	-	-	-	-	Fat-burning supplements not recommended.<br><br>Protein from increased dietary sources or supplemental isolated proteins has small but significant effect on<br>- Enhancing fat mass loss<br>- promoting retention of lean mass<br><br>Pyruvate - Small-to-trivial effect.<br>Chromium potentiating biological actions of insulin – Not recommended.<br>Green tea (polyphenol catechins and caffeine) as a thermogenic agent - Small-to-trivial effect<br>Alpha-Lipoic acid possible antioxidant - Small-to-trivial effect<br>Conjugated linoleic acid (CLA) for enhancing fat oxidation - Small-to-trivial effect<br>Konjac fibre (glucomannan) as water-soluble polysaccharide-dietary fibre - Small-to-trivial effect<br>Omega-3 polyunsaturated fatty acids possible appetite suppression, improved blood flow, and/or modulator of gene expression - Small-to-trivial effect	-

STUDY	CALORIES			CARBOHYDRATES			PROTEIN			FATS			MICRONUTRIENTS			SUPPLEMENTS	FLUID
	TIMING	TYPE	TOTAL	TIMING	TYPE	TOTAL	TIMING	TYPE	TOTAL	TIMING	TYPE	TOTAL	TIME	TYPE	TOTAL		
																Chitosan to reduce lipid absorption - Small-to-trivial effect.	
Burke et al. (2019a)	-	-	Focus on adapting energy balance (dietary energy intake and total daily energy expenditure) to support changes in body composition.	-	-	-	Periodize protein intake for physique manipulation.<br><br>Consume four to five protein eating occasions per day.	Consume high-quality protein-rich foods (high in leucine).<br><br>Protein-rich whole food sources are the preferred source of protein due to cost, safety and nutrient content.	Do not set protein targets to achieve the absence of insufficiency as this is not appropriate for competitive athletes who need to achieve a certain physique.<br><br>Increase emphasis on protein intake in situations of weight loss and when looking to retain or increase muscle/lean mass (about 1.6 - 2.4g/kg/day) alongside engaging in resistance exercise.<br>These higher end protein intakes do not increase risk of kidney problems or poor bone health.<br><br>Aim for a protein target of 0.4-0.5 g/kg/meal in the case of mixed meals that slow the protein digestion/absorption kinetics and scenarios of energy deficit/weight loss in which rates of muscle protein synthesis are suppressed.	-	-	-	-	-	-	Protein supplements may provide a valuable option when it is impractical to transport, prepare, or consume food sources of protein (e.g., immediately postexercise).	
Burke et al. (2019b)	-	-	Aim for brief periods of controlled low energy availability within the endurance training framework.<br><br>Avoid chronic or severe low energy availability, known as relative energy deficiency in sports, as this can cause health problems.	-	Consider a low fibre-diet in the days before weigh-in to reduce bowel contents and potentially a small loss of body mass.<br>The optimal period of implementation of the prerace low-fibre diet ranges from 24 to 72 hr depending on individual gut transit times.<br>- Experiment with the duration of the low-fibre diet to determine an optimal plan according to their usual fibre intake, gut transit time and personal tolerance of limited food variety, and reduced	- -	-	-	-	-	-	-	-	-	-	-	-

STUDY	CALORIES			CARBOHYDRATES			PROTEIN			FATS			MICRONUTRIENTS			SUPPLEMENTS	FLUID
	TIMING	TYPE	TOTAL	TIMING	TYPE	TOTAL	TIMING	TYPE	TOTAL	TIMING	TYPE	TOTAL	TIME	TYPE	TOTAL		
					satiety/hunger.<br>- Consider integrating with a carbohydrate-loading protocol.<br>- Meals and snacks should be based on low-fibre, CHO-rich foods<br>- Avoidance of significant sources of resistant starch.<br><br>Suitable foods include the following:<br>- White bread,<br>- White breakfast cereals (e.g., rice puffs),<br>- Sweetened dairy products,<br>- White rice, pasta, noodles, and potato: these should be well cooked and consumed hot to avoid the creation of resistant starch with cooling,<br>- Pulp-free fruit juice and sugary drinks (e.g., soda),<br>- Confectionary, jelly preserves, and honey,<br>- Cakes and desserts based on white flour (e.g., cakes, puddings) and sugar (e.g., Jello)<br>- Sports products (e.g., sports drinks, gels, confectionary)<br>-Meat, milk, cheese, poultry, fish, eggs, and other protein-rich foods can be added to meals and snack menus.<br>-Avoid uncooked fruits, fresh or dried, and vegetables, especially where they contain skin or pips. Cooked versions can be added in modest amounts to make up meals or menu items;												

STUDY	CALORIES			CARBOHYDRATES			PROTEIN			FATS			MICRONUTRIENTS			SUPPLEMENTS	FLUID
	TIMING	TYPE	TOTAL	TIMING	TYPE	TOTAL	TIMING	TYPE	TOTAL	TIMING	TYPE	TOTAL	TIME	TYPE	TOTAL		
					these include pureed fruit and apple sauce, mashed/ pureed vegetables with a preference for 'ketchup' style sauces and canned/ mashed vegetables												
Close et al. (2019)	-	-	Avoid a chronic low energy availability, as this is a major risk factor for bone injuries.<br><br>Cycle energy intake throughout the year to allow race weight to be achieved, while achieving adequate energy availability away from competitions, may be the most effective strategy.	-	-	-	-	-	-	-	-	-	-	-	-	-	-
Jager et al. (2019)	-	-	-	-	-	-	-	-	-	-	-	-	-	-	-	Probiotic supplementation is not recommended for altering body composition in athletes.	-
Lis et al. (2019)	-	-	Insufficient evidence to support fasting compared with conventional techniques for improving body composition.	-	-	-	-	-	-	-	-	-	-	-	-	-	-
Melin et al. (2019)	Aim to periodize energy and fuel availability around key training sessions.	-	Avoid long-term low energy availability (whether inadvertent or purposeful) as this may impair health and performance.<br><br>Aim for an energy availability of at least:<br>- 45 kcal/kg FFM/day for sedentary eumenorrheic normal weight women<br>- 40 kcal/kg FFM/day for exercising men appears to be a threshold to ensure optimal EA for physiological functions.<br><br>Avoid even short periods of energy availability (5 days) of less than	-	-	To maintain lean body mass and RMR during periods of energy restriction, adequate carbohydrate availability should be encouraged.	During periods of subclinical LEA, evenly distributed intake of protein (20-30 g every 3-4 hours) may support the maintenance of FFM and improve satiety.	-	To maintain lean body mass and RMR during periods of energy restriction, adequate protein intake (1.6-2.4 g protein/kg/day) should be encouraged.<br><br>Aim for intakes of protein of 20-30 g every 3-4 hours.	-	-	-	-	-	-	-	-

STUDY	CALORIES			CARBOHYDRATES			PROTEIN			FATS			MICRONUTRIENTS			SUPPLEMENTS	FLUID
	TIMING	TYPE	TOTAL	TIMING	TYPE	TOTAL	TIMING	TYPE	TOTAL	TIMING	TYPE	TOTAL	TIME	TYPE	TOTAL		
			30 kcal/kg FFM/ day as this impairs health.<br><br>Short periods of subclinical low energy availability may be necessary to reach the desired physique goals especially close to the competition season.<br><br>Use medical supervision including the use of sports dietitian when intentionally manipulating energy availability.														
Slater et al. (2019)	-	-	Avoid low energy availability when trying to reduce body mass.<br><br>Restrict energy intake with appropriate caution to ensure athlete health and performance are not compromised.	-	Insufficient evidence to use low-residue diets to cause weight loss and improve power-to-weight ratio in isolation in a sporting context.	-	-	-	-	-	-	-	-	-	-	-	Consider intentional dehydration as an alternative approach to acutely promote sufficient weight loss to favourably influence power-to-weight ratio.
Stellingwerff et al. (2019)	-	-	Macro periodisation of energy availability is needed to optimise body composition periodisation.	-	-	-	-	-	-	-	-	-	-	-	-	-	-
Tiller et al. (2019)	-	-	Careful consideration of weekly energy requirements of both training and recovery is recommended to achieve an individual goal of weight loss or gain.	-	-	-	-	-	-	-	-	-	-	-	-	-	-
Witard et al. (2019)	-	-	Aim to restrict energy intake and/or modestly increase energy expenditure to achieve weight or fat mass loss.<br><br>Aim for an energy deficit of approx. 500-1,000 kcal/day, for a weight loss of 0.5-1 kg/week.<br><br>The energy deficit required for weight loss is less during the initial stages of dieting than after 2-3 weeks.<br><br>As a result, the	-	-	Make subtle adjustments to both carbohydrate and fat intakes (approx.20% of total energy intake) rather than excessively reducing either macronutrient alone when increasing the protein content of an energy restricted diet.<br><br>Avoid over reducing dietary carbohydrate intakes to levels that may adversely affect the health and performance	Distribute protein evenly and frequently in relation to exercise and recovery.<br><br>Spread protein intake across four or five servings (i.e., three meals and one or two snacks, including a bedtime snack).<br><br>Consume protein sources postexercise to trigger a rise in MPS during weight loss.	Choose high-quality protein foods.<br><br>Emphasize leucine in protein sources consumed postexercise to trigger a rise in MPS during weight loss.<br><br>Consume food-based protein sources due to their nutrient density, which would benefit those who are on energy-restricted diets.	To achieve high-quality weight loss, ensure an adequate dietary intake combined with additional strength training as stimuli for lean tissue growth.<br><br>Ensure careful planning of dietary protein intake.<br><br>Aim for dietary protein intakes above daily requirements of 0.8-1g/kg body mass/day as this benefits manipulating body composition.<br><br>Strength/power or endurance-based track and field athletes with the goal of preserving	-	-	Consider small adjustments to both their carbohydrate and fat intakes (approx.20% of total energy intake) rather than excessively reducing either macronutrient alone when increasing the protein content of an energy restricted diet.	-	-	-	Protein-based supplements, including BCAA, for use on body composition during energy restriction is not recommended.	-

STUDY	CALORIES			CARBOHYDRATES			PROTEIN			FATS			MICRONUTRIENTS			SUPPLEMENTS	FLUID
	TIMING	TYPE	TOTAL	TIMING	TYPE	TOTAL	TIMING	TYPE	TOTAL	TIMING	TYPE	TOTAL	TIME	TYPE	TOTAL		
			degree of the energy deficit needs to be individualized.<br><br>Avoid long periods of energy deficit as this can impair health and performance caused by relative energy deficiency.<br><br>Avoid a severe energy deficit that requires a fast rate of weight loss as this is associated with a more pronounced loss of lean body mass.			of the track and field athlete.			or increasing lean body mass during weight loss should consume a dietary protein intake between 1.6 - 2.4g/ kg body mass/ day.<br><br>Athletes should target a daily protein intake between 2.2 - 2.4g/ kg body mass/ day if the athlete:<br>- habitually consumes a high protein diet<br>- possesses a significant volume of muscle mass<br>- has a high intrinsic metabolic capacity to degrade amino acids and generate urea<br>- selects a more aggressive weight loss strategy.<br><br>Aim for a protein intake per meal approx. 0.4 - 0.5 g/kg body mass or approx. 32 - 40g of protein expressed on an absolute basis for three meals and the remainder of protein as snacks.<br><br>Consider the habitual protein intake of the athlete prior to energy restriction, when setting the target level of protein intake during weight loss.								
Konig et al. (2020)	-	-	Avoid being on a long-term, low-calorie diet to lose body weight (e.g. in sports where weight is critical, such as ski jumping, gymnastics or dance) as this could compromise nutrient intake.<br><br>Aim to restrict energy and increase in protein intake in the short-term, to lose weight (reduction of fat mass) and keep loss of muscle mass to a minimum.	-	-	-	Aim to consume proteins several times a day (3-4 times per day).	Adequate quality of protein intake can have a positive effect on improving body composition.	Adequate quantity of protein intake can have a positive effect on improving body composition.<br><br>Increasing protein intake in the diet may be helpful for sports where periodic phases focus on increasing muscle mass or reducing fat mass.<br><br>A short-term increase in protein intake can sometimes be useful for planned weight loss (reduction of fat mass) through energy restriction that aims to keep loss of muscle mass to a minimum.<br><br>Aim for protein doses of up to 2 g/kg body weight per day.<br><br>During planned weight reduction while maintaining muscle mass, protein supply	-	-	-	-	-	-	-	-

STUDY	CALORIES			CARBOHYDRATES			PROTEIN			FATS			MICRONUTRIENTS			SUPPLEMENTS	FLUID
	TIMING	TYPE	TOTAL	TIMING	TYPE	TOTAL	TIMING	TYPE	TOTAL	TIMING	TYPE	TOTAL	TIME	TYPE	TOTAL		
									can be temporarily set higher.								
Wells et al. (2020)	-	-	Avoid inadequate energy availability of below 30 kcal/kg FFM/day in women and 20-25 kcal/kg FFM/day in men as this can impair bone health	-	-	-	-	-	-	-	-	-	-	-	-	-	-
Abreu et al. (2021)	-	-	During offseason, adjust total calorie intake to prevent weight gain.	-	-	-	-	-	Current recommendations for protein intakes during weight loss in athletes were set at 1.6 - 2.4g protein/ kg/ day.<br><br>Consider those athletes at risk of not achieving total protein requirements, including those trying to reduce their energy intake to control body fat mass.	-	-	-	-	-	-	Fat burners are not recommended.	-
Burke et al. (2021)	-	-	Aim for adequate energy availability to support usual training goals and dietary practices while achieving their weight class.	-	-	-	-	-	-	-	-	-	-	-	-	-	Consider a 2-3% BM reduction through acute restriction of fluid intake and/or promotion of sweat loss through exercise to levels commonly observed in routine training. Ensure aggressive recovery strategies are implemented after weigh-in.<br><br>Use of these practices to facilitate larger amounts of weight loss (>3% BM reduction) may have substantial negative implications to health and performance.
Collins et al. (2021)	-	-	Manipulate energy intake to elicit changes in fat mass or skeletal muscle mass.<br><br>Aim for an energy deficit.<br><br>Avoid a low energy availability, associated changes in training volume/intensity without	-	-	Daily carbohydrate intake of 4-8 g/kg body mass supports preseason training that aims to manipulate body composition (weight loss and fat loss).<br><br>Lower carbohydrate intakes may be needed where players are aiming for body fat loss	-	-	During energy restriction, protein requirements are likely increased due to the catabolic milieu created by an energy deficit.<br><br>A higher protein intake is recommended at 2.0 - 2.4g/kg BM/day, which depends on training load and weight loss.	-	-	Avoid over-restricting fat intake to <15%-20% of energy as this requires an unnecessary avoidance of a range of foods with valuable nutrient profiles.	-	-	-	-	-

STUDY	CALORIES			CARBOHYDRATES			PROTEIN			FATS			MICRONUTRIENTS			SUPPLEMENTS	FLUID
	TIMING	TYPE	TOTAL	TIMING	TYPE	TOTAL	TIMING	TYPE	TOTAL	TIMING	TYPE	TOTAL	TIME	TYPE	TOTAL		
			associated changes to fuelling.			(e.g., 4-6 g/kg body mass/day).											
Ferrando et al. (2023)	-	-	-	-	-	-	-	-	Whole-body essential amino acids requirements are higher with caloric deficit.<br><br>During caloric deficit, aim to meet whole-body essential amino acids requirements to preserve anabolic sensitivity in skeletal muscle.	-	-	-	-	-	-	-	-
Lowery et al. (2023)	-	-	-	-	-	-	-	-	-	-	-	-	-	-	-	-	Using coffee with athletes seeking body composition changes is not yet appropriate. Application to weight management in exercisers will require more consistent findings.
Sims et al. (2023)	-	-	Aim for a minimum energy availability of 30 kcal/ kg FFM/ day up until a threshold of 45 kcal/kg FFM/day.<br><br>Plan periodization of changing energy availability status to achieve optimal body composition.	-	-	-	-	-	-	-	-	-	-	-	-	Creatine is recommended for use in post-menopausal females.<br><br>Aim for a higher dose of 0.3 g/kg/d.	-
Leaf et al. (2024)	-	-	-	-	-	Consider the use of a ketogenic diet (defined as a daily dietary carbohydrate intake of less than 50 grams per day and a serum ketone levels above 0.5 mM) as it may cause greater losses in body weight, fat mass, and fat-free mass, when compared to a diet higher in carbohydrates and lower in fat. A ketogenic diet may also heighten losses of lean tissue, likely due to differences in calorie and protein intake, as well as shifts in fluid balance.	-	-	-	-	-	-	-	-	-	-	-
Number of papers (total 73)	8	8	45	2	7	19	9	8	24	0	5	13	0	4	10	23	9
